# Designing and modeling an IoT-based software system for land suitability assessment use case

**DOI:** 10.1007/s10661-024-12483-8

**Published:** 2024-03-19

**Authors:** Basma M. Mohammad El-Basioni, Sherine M. Abd El-Kader

**Affiliations:** Computers and Systems Dept, Electronics Research Institute (ERI), El-Bahth El-Elmy St. From Joseph Tito St., Huckstep, El-Nozha El-Gadeda, P.O. Box: 11843, Cairo, Egypt

**Keywords:** IoT, Land suitability assessment, Web development, Spatial–temporal dynamic

## Abstract

Assessing the quality of land is a very important step that precedes the planning of land use and taking management decisions; for example, in the agricultural field, it can be used to evaluate the suitability of the land for planting crops, determine the suitable irrigation system type, or adjust the agricultural inputs such as fertilizers and pesticides according to the requirements of each zone in the land. The spatial–temporal dynamic nature of land characteristics entails also updated evaluation process and updated management plan. The present paper tries to exploit the advances in information and communication technologies to develop a conceptual design of a dynamic system that accommodates the spatial–temporal dynamics of the agricultural soil characteristics to realize a land suitability assessment (LSA) based on a factor analysis method. The proposed design combines IoT technologies, web development, database, and digital mapping and tries to consolidate the system with other functionalities useful for decision support and suitable for different cases. The paper conducted a survey and made comparisons to select the best technologies that fit the current use case implementation and presents its reproducible conceptual modeling by developing the static and dynamic views through schemas, diagrams, message sequence charts, IoT messaging topic tree, pseudocode, etc. The functionality of the design was validated with a simple implementation of the system model. To our knowledge, there is no previous significant contribution that has addressed a LSA IoT use case. The proposed design automates the LSA process for more accurate decision-making, saving cost, time, and effort consumed in repeated field trips. It is characterized by flexibility and centralization in its offered services of spatial analysis, detection, visualizations, and status monitoring. The design also allows for remote control of field machinery.

## Introduction

The Internet of things (IoT) is the network of any physical object that contains sensors and/or actuators and has the capability to process and send data over the Internet. IoT represents a main pillar of technological revolution in nearly all the application fields: industry, agriculture, medicine, military, etc.

Therefore, IoT becomes the focus of attention of both research and industry. The literature is full of many proposals for IoT reference architecture and frameworks (Fremantle, 2015; Guth et al., 2016; Ngu et al., 2017; Saemaldahr et al., 2020; Abd El-Kader and Mohammad El-Basioni, 2013; Mohammad El-Basioni and Abd El-Kader 2020), IoT enabling wireless technologies development, IoT messaging protocols (Al-Masri et al., 2020; Naik, 2017; Tournier et al., 2021), and replete with many IoT use cases. In Srinivas et al.’s (2018) study, a use case was proposed for employing IoT technology in developing a smart healthcare system that contains an intelligent medicine box associated with sensors and a server for regular health monitoring. This smart medicine box with wireless Internet connectivity helps patients to get regular healthcare and creates easy communication between doctor and patient without meeting physically. The proposed medicine box helps the patient to take the right medicine at the right time.

In Zhang et al.’s (2018) study, an architecture for the Internet of intelligent things in smart hospitals was proposed, and an infusion monitoring system case study to monitor the real-time drop rate and the volume of remaining drugs during the intravenous infusion was developed. An automatic real-time system for automated vehicle parking based on IoT was proposed by Patil et al. (2020). In the field of entertainment, IoT was used to understand the foot traffic of people at events; this IoT application visualizes the attendee traffic patterns in real time to help sponsors understand the best places to advertise and to ensure that the attendee count stays within the fire code compliance of the venue (Mendix Technology, 2021).

IoT plays a key role in precision farming, as it allows continuous deep remote analysis, understanding, and control of agricultural processes leading to agriculture sustainability. This is clearly evident in the existence of various use cases for IoT in precision farming. IoT-based applications were developed for remote monitoring of the field information (Trimble Inc, 2021; Corizon, 2021; AgrIOT, 2021; Abd El-Kader and Mohammad El-Basioni 2021).

In Shylaja et al.’s (2021) study, an IoT-based smart device is proposed for crop monitoring using machine learning. Instead of using the classical learning schemes, it introduced a new scheme called Modified Learning based Field Analysis Strategy (MLFAS) that predicts the status of the crop in the field by analyzing the real-time input data acquired and reports this to improve the decision-making process. The work of Kaburuan et al. (2019) represents an IoT system for monitoring the performance of indoor micro-climate horticulture based on farmers’ treatment from planting until harvest time. The soil, water, air, and image data from the field were integrated with the weather data of the Indonesian Meteorological Agency data.

Just as the IoT is used to monitor climate and crops, it is also used for cattle monitoring where it senses and conveys data about the environment and cattle health. This application takes advantage of real-time data in the early treatment of any critical condition that threatens the cattle health and life before the matter worsens and disease spreads, for example. In Suresh and Sarath’s (2019) study, the proposed cattle monitoring system is composed of data-gathering nodes implanted on the cattle and contains sensors to measure important health parameters such as temperature, humidity, heart rate, and fall frequency. The system includes a mobile node that acts as a gateway that receives data from the data-gathering nodes and sends it to the IoT cloud platform for analysis.

The monitoring system proposed by Shabani et al. (2022) also senses the important health parameters of the cattle: body temperature, heart rate, humidity, and location. The main contribution of the system is a microservice-based architecture which works as a bridge between the implanted devices and the IoT cloud application, uses machine learning to produce a percentage for the health of each head of cattle, and notifies the farm manager of critical situations in real time. Other complete systems (Dutta et al., 2022; Trivedi & Chatterjee, 2022; Unold et al., 2020) have been developed to monitor cattle health including the monitoring devices, the cloud system, and the end-user application, differing in the types of sensors, the communication technology, the application features, and the algorithm used to analyze data, classify activities, and detect conditions.

IoT technology is used for remote control of farms, just as it is used for remote monitoring. Hence, it is used to automate sustainable agricultural systems and support their sustainability. Perhaps, one of the most sustainable agricultural systems that can benefit from IoT is the aquaponics system where it needs intensive monitoring and control. IoT-enabled aquaponics systems were proposed in the literature; in Haryanto et al.’s (2019); Hassoun et al. (2023) and Ezzahoui et al.’s (2022) study, smart aquaponics systems were proposed that monitor and control the water temperature, level, acidity degree, and fish feed using the readings of a number of sensors such as ultrasonic sensor, pH sensor, and temperature sensor. In addition to this, IoT has been widely used to monitor and control the environmental parameters in greenhouses (Joseph et al., 2020; Padmini et al., 2019; Shamshiri et al., 2021).

Regarding the agricultural systems which depend on management zone delineation and spatial analysis, few researches have talked about exploiting IoT technology in such systems. For example, Kang et al. (2012) suggested using IoT to deliver the data mashup which is used in zone management to produce intelligent decision-making, and they drew a simple conceptual system architecture for such a system without giving a specific implementation. In a research by Monteleone et al. (2020) exploring the factors affecting the adoption of precision agriculture for irrigation management in the context of Agriculture 4, in an experimental case study, the use of IoT was casually mentioned for implementing variable rate irrigation and zone management by producing irrigation prescription map, but also without details of the specific implementation.

This paper talks about a specific IoT use case which is the land suitability assessment (LSA). With the importance of LSA in achieving sustainable land-use planning especially in crop management practices, to the best of our knowledge, no or a small number of researches have addressed the exploitation of IoT to improve this process using the real-time data transmitted by the sensing devices deployed on the field. For example, the work proposed by Vincent et al. (2019) addresses the replacement of manual data collection with IoT-based sensors for LSA, but it mainly concentrates on the design of the multiclass classification used in land assessment. It only stores the data in the Amazon Web Service (AWS) cloud platform where it is used to develop the classification model which can be used later for future assessments.

The proposed conceptual design is for a complete software system for IoT-based LSA ready to receive data that comes from the field and stores and processes it based on a standard multi-criteria classification method. Also, it implements other functions such as spatial analysis. It is the generalization of our case study proposed by Mohammad EL-Basioni et al. (2022).

The Food and Agriculture Organization of the United Nations (FAO) had proposed a standard framework that specifies the procedures of the land suitability assessment process. In general, the process incorporates three steps. The first step is to select the influencing factors and grade the weights and relative values for the factors. The second step is to integrate the maps and database in GIS. Finally, the suitability score of each land segment is calculated, and the land suitability/classification maps are generated with respect to the given use. The various land-use suitability maps of a land are essential tools for planners and decision-makers to take the most suitable decision for this land use, management, and selection to allocate with the pre-plan, especially when they are taking into account sustainable development and economic competitiveness (Mu, 2006).

The multi-criteria land suitability analysis generic model can be expressed as a land suitability measure, ${\text{LS}}$, computed by a function of several indicators as shown in Eq. (1):

1 $${\text{LS}}=f\left({indicator}_{{\text{1}}},{indicator}_{{\text{2}}},\dots \dots ,{indicator}_{{\text{n}}}\right)$$

These indicators have a cumulative effect on land suitability as well as a separate effect that should be analyzed (Mendoza, 1999). These indicators are selected based on their sensitivity to management practices, ability to describe major soil processes, ease and cost of sampling and laboratory analysis, and significance (Tesfahunegn, 2014). These indicators are indexed in terms of some of their related soil parameters, and rating scores are given to these parameters.

The proposed system concentrates on land evaluation for sustainable crop production which depends mainly on the soil parameters and quality indices: fertility, chemical, and physical. The proposed system is built on the aforementioned approach, based on the parameters used and the model and formulas proposed by El Baroudy (2016). The used parameters include nitrogen (N), phosphorus (P), potassium (K), zinc (Zn), organic matter content (OM), soil salinity (S), the exchangeable sodium percentage (ESP), the carbonate (CaCO3) content, soil pH (PH), drainage (R), the texture (T), the soil depth (D), the slope (F), the surface stoniness (Y), the hard pan depth (HP), the hydraulic conductivity (G), and the water holding capacity (WHC). Some of these factors are related to the soil fertility properties, and some others are related to the chemical and physical properties of the soil. The criteria or the parameters used to measure each index can be varied; that is, some of the parameters considered in each index formula can be excluded, and the formula is modified accordingly.

The variability in soil properties, especially over large areas, and the change in soil over time have made the owners of large-scale farms to periodically analyze soil to test its health and determine the most appropriate treatment and use. These large-scale farms resort to the variable rate application technology to optimally deal with this variability, where it allows adjusting the amount and rate of agriculture resources throughout the farm according to each specific region’s needs without excess or negligence that harms the crop and without wasting resources and harming the environment. The data from soil analysis is used to produce a prescription map which guides the variable application.

A variable rate irrigation system is used in farms which have center pivot systems. According to the prescription map, the center pivot control system cycles control the switching of sprinklers and vary the speeds to achieve the desired application rates in each section of the farm.

The farms which have tractors with onboard computers and adjustable rate spreaders, seeders, or sprayers can be used in variable rate fertilizer application, variable rate seeding, or variable rate pesticide application, respectively, by loading the prescription map to the computer.

Using IoT technology in the generation of prescription maps maximizes the benefit of the variable rate application by improving spatial resolution and addresses the issue of soil change over time thus improving temporal resolution as well. Therefore, these large-scale farms with computerized agricultural application equipment are potential customers for IoT-based systems for spatial analysis and management zone delineation in variable rate application, hence the proposed system.

One of the most important tasks of scientists and technical specialists involved in agricultural planning is land-use planning, especially the LSA process. The functionality provided by the proposed system that allows the LSA maps to be generated automatically from real-time data and stored historical data makes it a desirable and very useful tool for these scientists.

The rest of this paper is organized as follows, the “Materials and methods” section identifies the technologies that were used to build the system and the tools that were selected to implement these technologies, and then, it formulates the IoT-based LSA use case design including its static and dynamic view. The “Results” section represents a small-scale implementation of the proposed design to validate the functionality and suitability of the selected technologies and tools to the system use case. The “Discussion” section discusses the work presented in this paper and the development of IoT use cases for farm enterprises in general. Finally, the “Conclusion and future work” section concludes the paper and specific directions for future work.

## Materials and methods

This section reviews the selection of technologies and tools for system implementations and contains system modeling.

### Selection of technologies

The main technologies which build the proposed system are web application development, digital mapping, database, and IoT. The following sub-sections review recent trends in these technologies and set criteria for technology selections for the system implementation.

#### Web application technologies

The web application user can access it through the web browser where he can use its functions and perform some tasks that need interactions with a remote server over a network, which can be the Internet, using a client–server connection paradigm. Accordingly, developing a web application incorporates two types of development: front-end development and back-end development. The front-end development, or the client-side programming, is responsible for all the elements and layout the user directly interacts with on his browser. The back-end development, or the server-side programming, is behind-the-scene functionality that enables the front-end functionality; it represents everything the user does not see or directly interact with, but in fact, it powers what is happening.

##### Front-end development

Mostly, there will not be a front-end webpage that can dispense with the presence of the HyperText Markup Language (HTML) and Cascading Style Sheet (CSS) coding. HTML is the organizer of the page structure, and CSS is responsible for its component formatting and styling.

To make the front-end webpages dynamic, a programming language is needed, and JavaScript is undoubtedly the most popular language used to do so. JavaScript is an event-based runtime scripting language that can use the HTML Document Object Model (DOM) to add animations, automatic refreshments, and adjustments according to events such as user input timers and also server-side events; therefore, it supports the functionality of the webpage with which the dropdown lists, the buttons, the checkboxes, the tables, the menus, etc. of the webpage become functional and interactive with the users. In addition, JavaScript can fetch additional web content dynamically and run web-based software. Developing with JavaScript, we will generally need to use some JavaScript libraries, may be with templating engines or frameworks. Libraries are used to speed-up and facilitate development where they contain pre-written ready-to-use JavaScript functions; the two most popular JavaScript Libraries are jQuery (Vats, 2021) and React JS (JavaTpoint, 2021). Table 1 presents their important features which can be used for making a comparison to decide which one is more suitable for implementing the proposed system.

**Table 1:** Comparison between jQuery and React JavaScript libraries

	jQuery	React
Recent adoption trend	Decreasing curve	Increasing curve
Popularity	Still most popular than React in all countries. About 30.142% of the entire Web are using jQuery, according to SimilarTech	About 1.735% of the entire Web are using React, according to SimilarTech
Ease of use and developer preference	Easy to use, fast, lightweight, and feature-rich, low learning curve	Also, easy to use, but ReactJS uses JSX. It's a syntax extension that allows HTML with JavaScript mixed together. It may be considered a barrier due to its complexity in the learning curve (the learning curve for this framework is high). Generally, it keeps on changing with time which makes its learning more problematic, in addition to its poor documentation
End-user experience	Directly interacts with the DOM which results in a slightly lower performance than React. Its library size is just 75 KB, which is nearly 18% lower than React which makes the pages load faster improving the user experience	Uses virtual DOM for better speed, and performance, React has proved extremely demanding for applications that require dynamics, often upgrades and updates. While the size of its library, which is considered to be about 95 KB, affects adversely the user experience where it makes pages load slower than jQuery
Size and complexity of applications	It is suitable for small-size non-complex applications. It may cause spaghetti code	Due to its components-based architecture and virtual DOM, it is suitable for large-size and complex applications. The code is more predictable and easier to debug
Time-to-market	Short time-to-market with competitive results due to its simple syntax	It may result in longer time to develop pages due to complex UI with dynamic page content and controls. The application development time may also be affected due to the learning curve complexity
Cross-browser compatibility	It ensures that the webpages are rendered correctly regardless of the browser, and supports older browsers	It supports all popular browsers, including Internet Explorer 9 and above, although some global polyfills are required for older browsers such as IE 9 and IE 10
Websites use it	Uber, Twitter, Linkedin, Udemy	Facebook, Instagram, and Whatsapp

JavaScript frameworks are collections of JS libraries that not only include JS code snippets, but also provide developers with a template for arranging the JavaScript code on their website. They are scaffolds for building your front end. They usually include some way to structure the site files (for example, via components or a CSS preprocessor), make AJAX requests, style your components, and associate data with DOM elements.

There are other less common languages through which one can do front-end development depending on the framework, for example, Flutter uses Dart and Django uses Python. But nowadays, the most frameworks getting popular are Angular and Vue.js. Angular uses TypeScript programming language and offers an incredible structure, providing easy two-way data binding, an MVC model, a built-in module system, a routing package, dependency injection, and other exciting features. Vue.js is a progressive JavaScript framework used to build web interfaces and one-page applications. The HTML extension and the JS base made Vue a favored front-end tool; it is used by Adobe, Alibaba, Gitlab, and Xiaomi. Table 2 shows Angular’s and Vue.js’s important features.

**Table 2:**
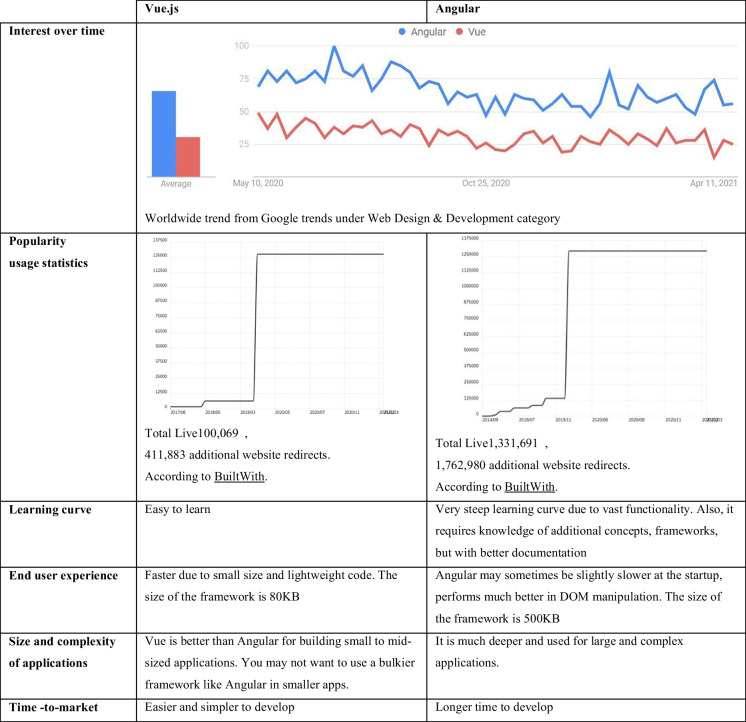
Comparison between Angular and Vue.js JavaScript front-end frameworks

Templating engine is a way to display information on the user’s browser. It aims to describe what we want to show. Templating engines such as Handlebars.js, Embedded JavaScript Templates (EJS), Underscore.js, and mustache allow to store HTML/CSS/JavaScript components and insert JavaScript variables into them.

**Fig. 1:**
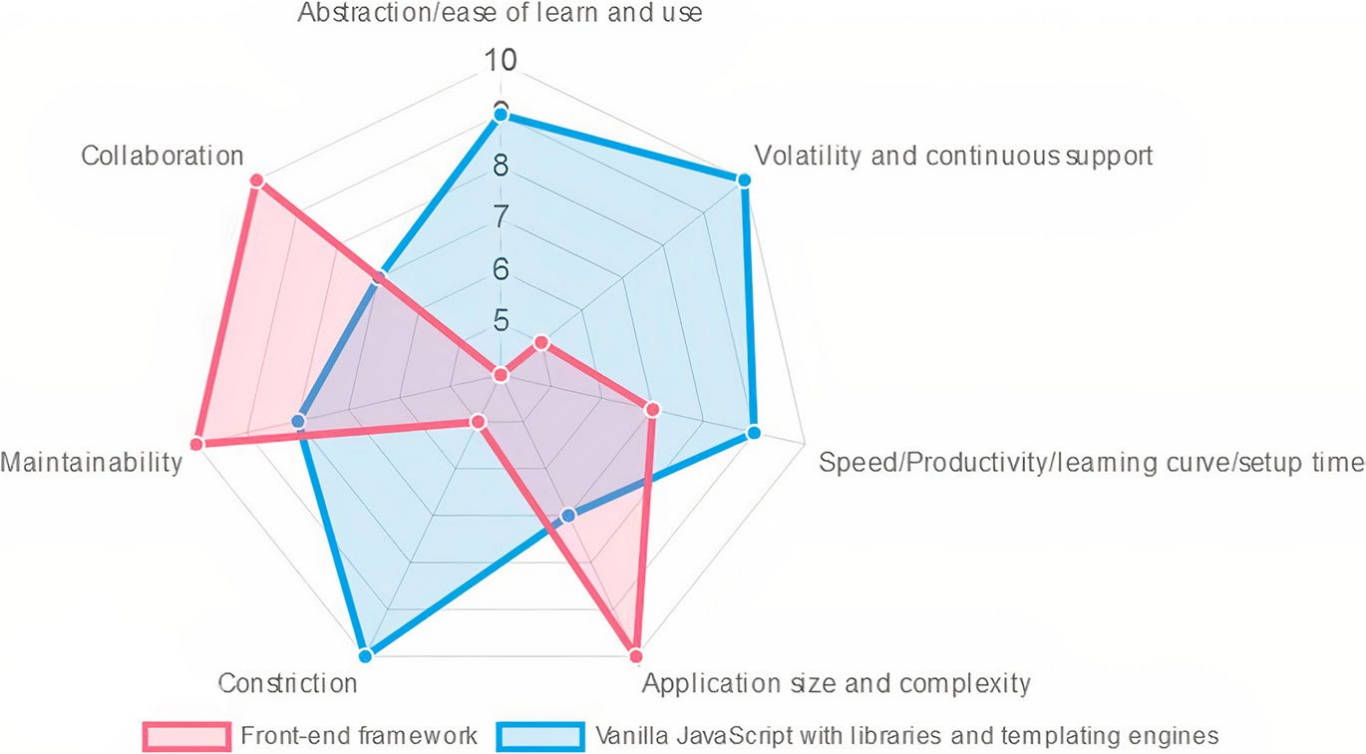
Ranking for the front-end development approaches in terms of some important development metrics

But does the web application of the proposed system need a front-end framework or is vanilla JavaScript with some libraries and templating engines as needed sufficient? Fig. 1 shows the relative ranking for these two methodologies in terms of ecosystem evolution rate and continuous support, constriction feeling in the design, productivity/learning curve, application size and complexity, maintainability, collaboration of developers and ease of understanding codes, and abstraction/ease of learn and use.

##### Back-end development

The traditional back end is a mix of four components: the server, databases, software, and operating systems. It includes the code that connects the web to a database and manages user connections and code that powers the web app itself.

Its job is to (1) access the information that users require through the application, (2) combine and transform such information, and (3) return the information in its new shape to the requester. Back-end development works in tandem with the front end to deliver the final product to the end user. The following sections talk about each back-end main component.

Application (middleware/server-side software/API): the application is the underlying code bridging the clients to the database and other back-end and cloud resources, it is what ultimately drives the web service. The most common back-end programming languages can be categorized into:

- *The Object Oriented Programming (OOP) languages*: languages which follow the imperative programming model that uses statements that change a program’s state. An imperative program consists of commands for the computer to perform. OOP is used when there are many things with few operations. Popular OOP languages are Java, JavaScript, TypeScript, ASP.NET, Python, and PHP.
- *The Functional Programming (FP) languages*: languages which follow the declarative programming model: a high-level program in which the coder “declare” the results that he wants the language tool to output without writing explicit commands, and the tool processes the declaration and produces the required output. The functional language is non-imperative. The state transitions are represented in pure functional language by functions; it treats all computations as the evaluation of mathematical functions. Functional is used only when there are few things with more operations (performing many different operations for which the data is fixed). Popular functional languages are SQL, F**#**, and R.

It is always up to the programmers or developers to choose the programming language concept that makes their development productive and easy. But what are the hypotheses for the selection? What are the language categories and the language to use in the system implementation?

First, for choosing the programming paradigm, as the choice is problem-specific, it is more likely to use a mix of the two approaches depending on the current problem at hand, which is the best way to implement it. In case of mathematical problems, FP can effectively implement it. In other cases, if a problem can be decomposed into a number of procedural steps and when performing many different operations for which the data is fixed, go functional; if, on the other hand, a problem involves groups of inter-related agents and when performing small operations for mutable data, an OOP approach is likely to be better.

**Fig. 2:**
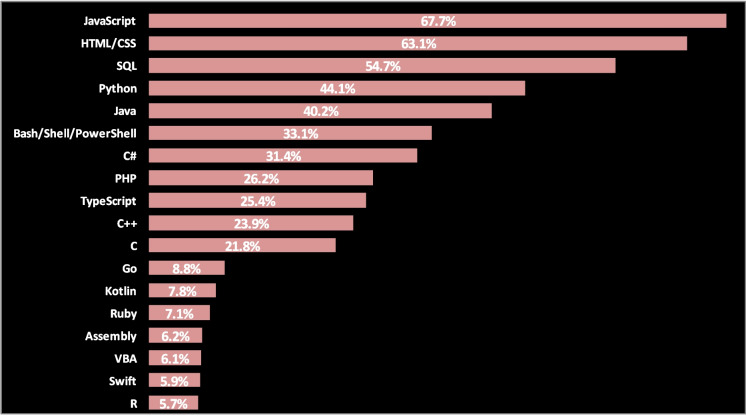
Stackoverflow developer survey for programming, scripting, and markup languages popularity

**Fig. 3:**
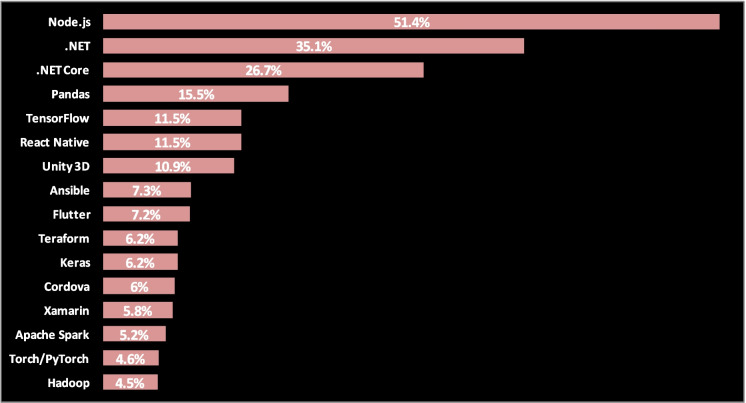
Frameworks, libraries, and tool StackOverflow trend survey

Second, what is the OOP programming language that will be used? The popularity is not the sole reason which makes us select the programming language, but it is a good indicator of the need of developers, who we are from them, to learn and use the language; therefore, the language popularity indicator accompanied by reviewing its application and features can be the rule for selection. According to the StackOverflow Developer Survey result 2020 (Fig. 2), JavaScript is the most commonly used OPP language and then Python and Java, C#, PHP, TypeScript, C +  + , and C. And according to frameworks, libraries, and tool StackOverflow trends up to the year 2020 (Fig. 3), Node.js takes the top spot as it is used by half of the respondents.

Node.js is technically not a language but a runtime environment for JavaScript with some specific useful libraries that can be used by JavaScript. It is better suited for building scalable, event-based, real-time, data-driven, I/O heavy, fast applications, accessing or performing any non-blocking operation of any operating system, like creating or executing a shell script, accessing any hardware-specific information, or running any back-end job. As it helps us to use JavaScript from outside of the browser, it enables JavaScript to be used in the back end as well as the front end and in any OS, solving JavaScript compatibility issues. In addition, its event-driven back-end nature enables it to handle multiple synchronous database read/write/update requests, bearing in mind that the IoT is characterized by the collection of a huge amount of data.

It is actually very common to use more than one back-end programming language, and there are a number of ways this can be done, for example, calling libraries that are written in another language or as separate processes. Node.js has different packages developed for interfacing with other languages or runtime environments. Node.js frameworks include:

- *Express.js*: This is a minimalist web application framework that is used to build a number of mobile applications and APIs.
- *Socket.io*: It is used for building real-time web applications. It is a JavaScript library that allows the bidirectional data flow between the web client and server. Asynchronous data I/O, binary streaming, and instant messaging are some of the most important features of this framework.
- *Derby*: This is a real-time data synchronization engine for Node.js that allows multi-site, real-time concurrency, and data synchronization across clients and servers.
- *Sails.js*: This framework has gained traction through the development of chat applications, dashboards, and multi-player games. It is most famous for building data-driven APIs.

Due to the previous analysis, we will concentrate on the Node.js technology.

Servers: they are responsible for managing all back-end processes such as exchanging data among different clients, managing databases, reacting to users’ posts, and sending emails. Rather than the need to have physical servers, now, cloud services, such as Amazon and Azure, provide virtual servers on which the developers deploy their server applications. In addition, with the Backend-as-a-Service (BaaS) cloud service model, the developers can concentrate only on developing the front end. The system includes servers:

- *Web server*: Node.js has a built-in module called HTTP that can create an HTTP server, while Express is a Node.js web application framework built on the HTTP module, adding more functions to what it offers to increase the convenience of building web servers.
- *IoT server*: Node.js is equipped with libraries that support IoT messaging protocols; node-coap is a client and server library for CoAP; rhea is used for developing AMQP client and server. MQTT protocol and socket communication which are commonly used in IoT applications are supported in Node.js: Mosca is a Node.js MQTT broker which can be used standalone or embedded in another Node.js application, and MQTT.js is a client library for the MQTT protocol.

**Fig. 4:**
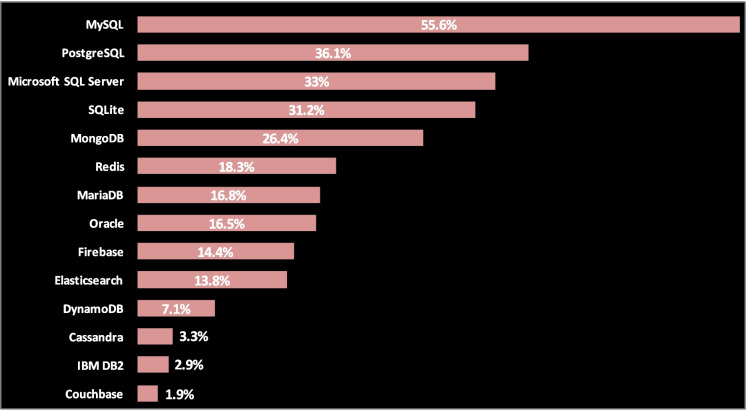
Stackoverflow developer survey for databases

Database: Node.js supports relational databases (e.g., MS SQL Server, Oracle, MySQL, PostgreSQL, SQLite) and NoSQL databases (e.g., MongoDB, Cassandra, LevelDB, Redis). BaaS databases can also be used allowing us to focus on developing the client-side, such as Firebase, Parse Server, Hoodie, Horizon, and feathers-nedb. Most BaaS providers use NoSQL databases. According to StackOverflow developer survey result 2020 for databases (Fig. 4), MySQL occupies the first rank in popularity among developers, followed by PostgreSQL and Microsoft SQL Server.

##### What to use in the proposed system front-end and back-end development

The proposed IoT-based system is not considered to be a large-size and complex application. By reference to Fig. 1, the frameworks are harder to learn especially since it is imperative to also learn the underlying code of the framework to use it better and for the purpose of customization and adding functionalities, and all of these increase the production time using frameworks. With respect to the constriction in the design, some frameworks are described to be opinionated, their design is based on principles that are not suitable to the application we want to develop or increase the application footprint by unnecessary unusable codes, and at the same time, their customization is a too tricky and nontrivial task; this raises the need to study the features of the available frameworks, compare them, and then select the appropriate one to study and use; this is also not an easy task and may result in an inaccurate choice.

All of these reasons in addition to the availability of a variety of JavaScript libraries and the guarantee of related good and continuous support, it is preferred to build the proposed system from scratch using the front-end design approach—vanilla JavaScript with some libraries and templating engines—by trading off the frameworks’ built-in functionalities, reliability, and maintainability. By the same token, jQuery is preferred over React due to easiness to learn and use, short time-to-market, better documentation, and other good features such as lightweight, feature-rich, and cross-browser compatibility.

Naturally, HTML and CSS, along with other HTML, CSS, and JavaScript-appropriate libraries, are essential for the proposed system front-end development. It is expected to use libraries and plugins for supporting specific helper functions such as Bootstrap CSS framework for getting design templates for some interface components; Font Awesome which is a font and customizable icon toolkit; JSZip for creating, reading, and editing.zip files; html2canvas for taking screenshots; SheetJS for spreadsheets manipulation; and Google Chart libraries.

The real-time, scalability, and concurrent processing and harvesting a vast amount of data are the main needs for precision agriculture and IoT applications, and all of these metrics are realized by Node.js; therefore, as indicated earlier, our choice fell on using Node.js to implement the back end of the system and interface it with other FP languages if needed. Using the same language—JavaScript—in both front and back ends is an advantage.

Relational database will be used to manage the proposed system’s structured data and represent the relations between them. Node.js can be interfaced with both of the two most popular database management systems MySQL and PostgreSQL. PostgreSQL is an object-relational database. While MySQL is simple and easy to understand and manage which improves its speed performance for simple applications, PostgreSQL is preferred for implementing the proposed system for supporting complex queries on large data volumes and due to its support for NoSQL features and a large number of data types including JSON.

**Fig. 5:**
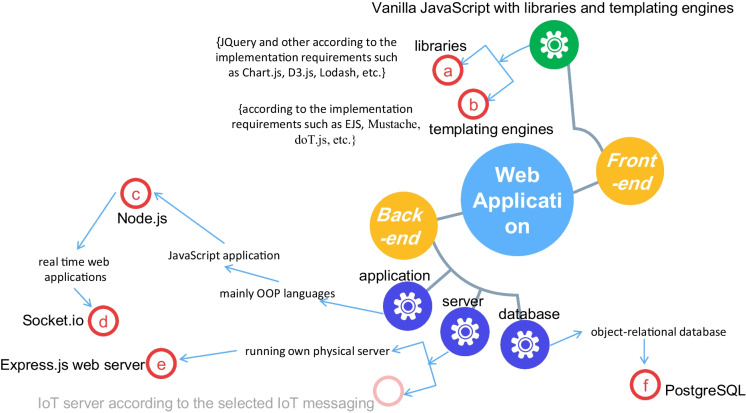
The selected web development technologies for the proposed system

Finally, the proposed system back end will use the Express web server. Figure 5 summarizes the system web app development technologies.

#### IoT technologies

The IoT will utilize the existing networking infrastructure, technologies, and protocols currently used in homes/offices and on the Internet. The IoT runs over the existing TCP/IP network infrastructure.

**Fig. 6:**
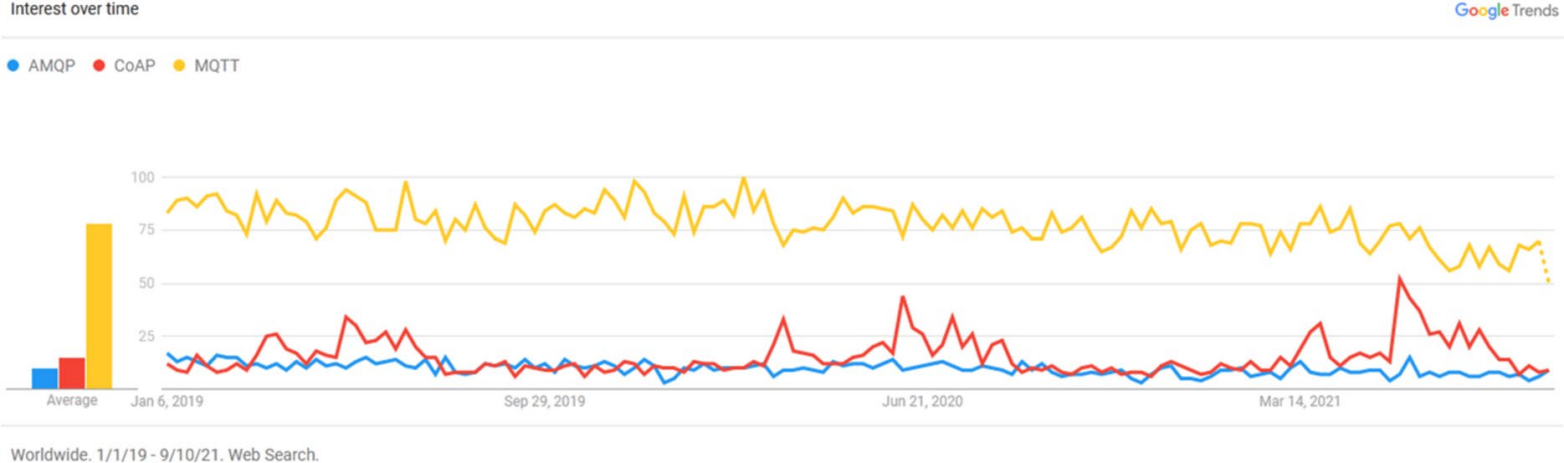
Google trends for three well-known IoT messaging protocols

Exchanging data through messages is fundamental for the development and deployment of IoT applications. The IoT protocols, or the IoT messaging application protocols, can take the publish/subscribe model or the request-response model, some of them use UDP and some others use TCP/IP. Figure 6 describes the trends in the worldwide interest in three popular messaging protocols: MQTT, CoAP, and AMQP.

The publish/subscribe model has a lot of characteristics and advantages that make it suitable for IoT scenarios especially the proposed near-real-time system case, including asynchronous many-to-many communication, decoupling between senders and receivers in time, space, and synchronization, reliability, and scalability.

**Fig. 7:**
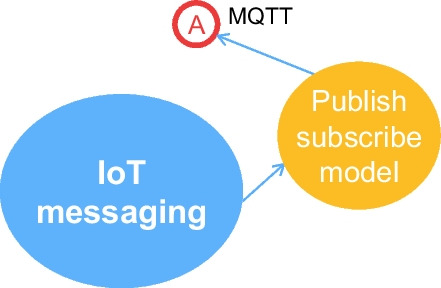
The selected IoT technology for the proposed system

The publish/subscribe paradigm is supported by both MQTT and AMQP; however, AMQP is heavyweight and therefore not suitable for battery-operated IoT devices, while MQTT is very lightweight. This is one of the main reasons why MQTT is the de facto standard for IoT messaging (HiveMQ, 2021) and more widely used than AMQP in IoT applications and the reason why we selected it as the adopted IoT technology for the proposed system (Fig. 7).

#### Digital mapping technologies

Google Maps is a feature-rich online mapping framework that supports numerous geospatial functionalities, and it is the first mapping service and API the developers think of choosing when integrating maps into a web application. It has earned the trust of web, iOS, and Android developers around the world. However, Google Maps increased the cost of its API calls—reaches $14 for 1000 map calls—and made the number of free API calls per month nearly 30 times less; this makes the search for alternatives a must. The two most prominent alternatives to Google Maps API are Mapbox which offers free 50,000 API calls monthly and $0.5 for each subsequent 1000 calls (Osypenko, 2021) and OpenStreetMap which is free.

**Fig. 8:**
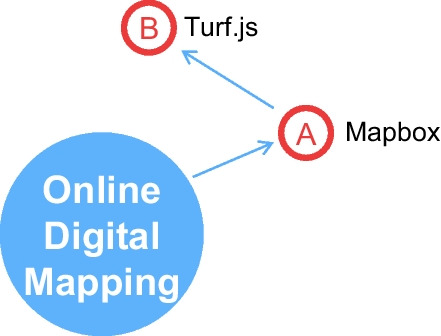
The selected digital mapping technologies for the proposed system

The OpenStreetMap is considered to be a great geographic database of the world, but it does not offer a lot of mapping functionalities, so usually, it is better to use a more powerful service built on its data such as Mapbox. Mapbox is one of the largest providers of maps for websites and mobile apps. Their service is used by GitHub, Pinterest, Foursquare, and other popular companies. Mapbox offers advanced features such as location search, route finding, augmented reality, and many tools to help in integrating maps and other Mapbox online web mapping services—such as Directions, Geocoding, and Static Images. Mapbox is characterized by custom maps, very good performance in loading maps, the ability to deal with a huge amount of data to visualize and extract conclusions to take appropriate actions, and geospatial mapping support. These features make it a good fit for the proposed system type. Turf.js is an open-source JavaScript library used to perform advanced geospatial analysis in browsers and Node.js, and works with Mapbox. It includes traditional spatial operations, helper functions for creating GeoJSON data, and data classification and statistics tools. Figure 8 illustrates the selected mapping technologies.

#### Measuring and transmission technologies

There are several methods to measure the soil parameters for computing the quality indices and assess land suitability. Some parameters can be sensed with MEMS sensors available commercially or proposed in the literature, on-the-go vehicle-based sensors, handheld measuring devices, or soil sampling and laboratory analysis. Returning to the parameters of soil fertility, chemical, and physical properties mentioned previously, we find that the parameters that can be measured by industrial sensors embedded in MEMS-based wireless deceives, deployed in the field, communicate, and send data in real time are N, P, K, S, WHC, D, PH, T, and F. The sensor can be an integrated sensor that measures more than one soil parameter or a sensor dedicated to measuring a specific parameter. The soil NPK sensor (Soil NPK Sensor 2023) can measure the fertility and nutrient parameters N, P, and K. The soil EC-moisture-salinity-temperature sensor (Soil EC-Moisture-Salinity-Temperature Sensor 2023) can be used to measure and compute S, WHC, and D. The soil nutrient sensor (SEM225 Series Soil 2023) can be used to measure S, WHC, D, along with the PH value. The RS-PH-N01-TR-1 (Soil PH Sensor 2023) is a sensor dedicated to measuring PH. Acoustic sensors can be used to measure T and D, and the soil slope/tilt sensor such as 0750–9002-99 MEMS tilt sensor and inclinometer (0750–9002-99 ± 90° RS-485 IP67 MEMS Tilt Sensor and Inclinometer 2023) and the dual-axis inclinometer (AXISENSE-2 V-OUT TILT SENSOR FLOOR MOUNT 2023) can be used to measure F. In the literature, a frequency-domain integrated sensor was proposed for in situ estimation of G (Xu et al., 2022), while the ring infiltrometer tool is already used for this. Optical sensors were proposed in the literature to measure OM, while commercially available handheld electromagnetic sensors, such as Geonics EM-38 Ground Conductivity Meter 2023, are used for infield measurement of OM as well as R and T. A handheld soil penetrometer is used to measure HP. The Zn is deduced from remote sensing data or from other influencing factors, such as the PH and T, Soil sampling and laboratory analysis can be used to provide information about Zn, HP, ESP, CaCO3, and Y.

The data reported by the battery-operated devices deployed in the field in such a system is not characterized by a high data rate, but the devices require low power consumption for a longer lifetime, and the long-range bidirectional secure communication and low cost are preferred. These requirements are satisfied by the unlicensed low-power wide-area network technology, especially LoRaWAN. LoRaWAN has long been used for agricultural and environmental monitoring; its communication range reaches 5 km in urban areas and up to 15 km in rural areas (LoRa and LoRaWAN 2023). LoRaWAN is composed of end devices handling the sensing and actuation and performing bidirectional LoRa communication with the gateways which connect them to a server. The server manages the network through different pieces of software used for authentication and authorization and session management, network configuration and performance monitoring, data routing and selection of the gateway for a downlink, handling uplink messages duplication, processing application data, encryption/decryption and integration of data into existing management systems or IoT platforms, etc.

### Formulating the IoT use case: system design 

This section tells the story, identifying the need, the problem, and the requirements, which motivate constructing a proposed system use case. Then, the section goes on to explain the static structure and the dynamic behavior of the system.

#### Narrative description of the system use case

Some study areas can be employed to produce most types of vegetables and fruits including potatoes. It is a large area of high spatial variation and is characterized by using modern cultivation methods such as pivot irrigation. These modern cultivation methods often are associated with the variable rate application of agricultural inputs to encounter and exploit the high spatial variability of the field phenomena. Assuming the existence of all the required hardware used in applying the variable rates whether built-in or added to the modern agricultural equipment, the challenge is in developing the plans that control how the rate should be varied in the different field regions which is usually based on geo-referenced sampling of field data. This process, to be accurately achieved, requires experience, may require the hiring of skilled persons to achieve it, and repeated modification of the plan requires more data collection throughout one season; therefore, it consumes a lot of effort, time, and money.

The proposed system use case uses the IoT technology and recent trends in programming and web development to enable the automation of data collection with higher temporal and spatial resolution, automate the spatial analysis and management zoning process, and facilitate, centralize, and improve decision-making.

The system depends on the existence of sensing devices deployed in the field in certain points with known geographical positions, takes periodical samples of the field phenomena, and reports their values in real time in addition to laboratory values determined after analysis of soil samples taken at the same positions by special different devices. These laboratory values are inputted to the system manually.

The system is prepared to manipulate field data related to soil, climate, and plant. All the data is stored in a database with its geographical coordinates for historical reference.

The real-time data along with the stored laboratory values are used to infer the values of phenomena along the field by interpolation, and then, each region which has the same range of values and accordingly indicates a specific performance and requires special management is delineated whether it is contiguous or spread over the field area, all of this using real maps of the earth.

Examples of management zone map usage include phosphorus and potassium management zone maps for variable fertilizer application, management zone maps for soil texture and soil water content can be used for variable irrigation, climate zone maps can be used for weed control, chlorophyll content measurement maps can be used for nitrogen management, and soil electrical conductivity maps can help for explaining yield variation.

Moreover, the system allows for the spatial analysis of soil quality indices of fertility, chemical properties, and physical properties. The spatial analysis of these soil quality indices is used to derive the land suitability assessment classes for crop production.

The appropriate decision is not limited only to the application in the appropriate place but also to the appropriate decision time so that the system should raise alerts immediately when certain conditions exist. Likewise, appropriate decision-making may need to be done based on reviewing historical data and representing it in different forms including management zone maps.

The system should be characterized by flexibility in modifying the modeling equations, parameters’ values, adding and removing items, selection from different options, and different types of user accounts with different privileges.

The system should be tailored for any land and not limited to only one field such that the user can manage all his belonging farms from his place through the application, apply to them all the system functionalities, and accordingly be able to compare their characteristics.

The maximum benefit of this system is when all the incorporated parameters are measured in near real time through the field devices, but as mentioned before, not all parameters have commercially available MEMS sensors to measure them. Some parameters are still measured by handheld devices or through laboratory analysis of soil samples, and their values are then entered into the system manually, for example, through spreadsheets, for use in analysis with continuous data received from the field nodes.

Referring to the “Measuring and transmission technologies” section, the sensor nodes deployed in the field will be LoRaWAN end devices of “class A,” each containing the four sensor types which measure the indicated real-time parameters. As outlined above, the equation used to calculate a soil quality index can be modified by omitting some parameters and modifying the exponent accordingly by decreasing its denominator by the number of omitted parameters. Thus, besides having one type of node containing all the sensors that are included in the analysis, there can be three types of nodes according to the embedded sensors used separately: Type1 sensor node which is a fertility sensing node containing soil NPK sensor, Type2 sensor node which is a physical property–sensing node containing tilt sensor and acoustic sensors, and Type3 sensor node which is a chemical/physical property–sensing node containing soil nutrient sensor.

The system is designed and configured to receive all values included in the LSA equation in the sensor node message payload, the actually sensed parameters will have values, and the unsensed parameters are marked with NULL. The parameters manually entered into the system are retrieved from the corresponding table, and likewise, NULL values are ignored in analysis calculations.

The sampling frequency is the same for all sensors in a single node and can be configured remotely. Whenever a new message is received from a node, its measured sensors’ data is stored in the appropriate tables accompanied by the reception time and date. The last record of each node is treated as the recent data from this node, while the system service that will be mentioned later “Nodes Status Monitoring panel” alerts if a node ceased sending for a specified time.

Thanks to the long LoRa range, the star-of-stars topology is satisfactory to such a system; any node deployed in a plot of agricultural land can reach a gateway deployed anywhere within that plot or beyond. Taking into account its minimum communication range, it can be said that an outdoor LoRa gateway, which costs an average of $600, can serve a circular area equal to more than 18,000 feddan. The appropriate number of nodes for a given field can be determined depending on the soil sampling scheme used. For example, if grid sampling is used, at least one node might be placed in the center of the cell or at a random position within the cell. The approximate price range for the soil NPK–integrated sensor is $28–95, and the price of the 4-in-1 soil nutrient–integrated sensor is $51. The tilt and acoustic sensors are cheaper especially when a quantity is purchased. A microcontroller board with a LoRa transceiver costs about $20. The sensor node contains other components such as a GPS module and batteries. Taking this into consideration, it can be said that the cost of such a sensing station containing all the sensors is no more than $340.

As mentioned above, the system use case model is adapted to the fact that some phenomena still do not have corresponding MEMS sensors, the flexibility required in modeling soil quality indices, different types of sensors, different sampling frequencies of nodes, the possibility of one or more devices stopping transmission, and different measurement scales by mapping the parameter values to a specific set of factor scores provided by the land suitability assessment model as will be illustrated later.

#### The static view of the system

**Fig. 9:**
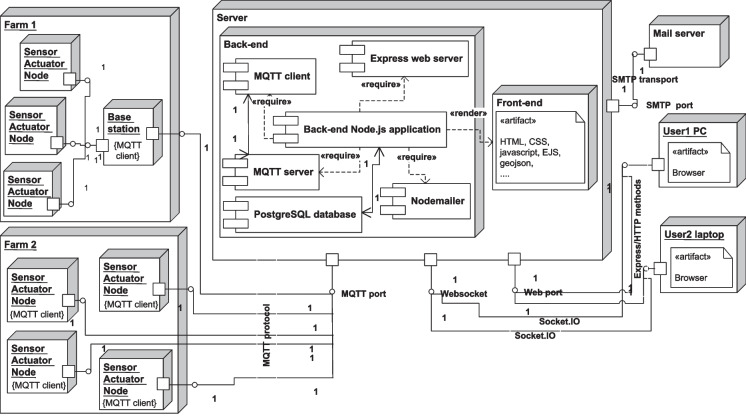
Deployment diagram of the proposed system

The topology for the system hardware and artifacts is summarized in the UML deployment diagram depicted in Fig. 9 which shows the system nodes including a central physical server consisting of back-end and front-end artifacts. The core of the back end is the Node.js application which loads the modules required for the server functionalities, mainly, the Express web service, the Nodemailer for email sending, and PostgreSQL client modules for connection to the PostgreSQL database with a specified connection configuration. The back-end works as an MQTT server as well as an MQTT client; the back-end MQTT client connects to the MQTT server using the MQTT protocol. Likewise, the system nodes include instances for user devices which connect to the system back end through HTTP and WebSocket, and instances for the IoT devices in different farms represented by MQTT clients reside on the sensor actuator nodes or on a base station which sends the data of the sensor actuator nodes on behalf of them, forwards the configuration, and commands messages to them.

#### The dynamic view of the system

**Fig. 10:**
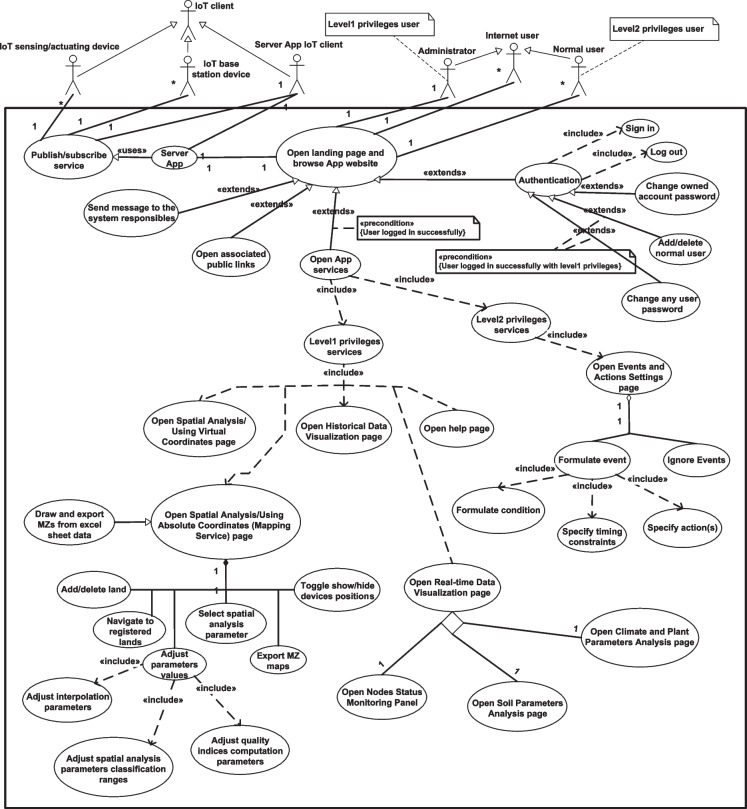
The proposed system use case diagram

In the following, the dynamic behavior of the system will be modeled, starting by describing the functionalities and the high-level design requirements of the system including actors influenced by the use case diagram in Fig. 10. There are about thirty-eight use cases and seven actors associated by different relationships. The administrator and normal user actors are generalized to their parent actor, the Internet user, and they are associated with use cases specific to their roles with two levels of privileges that require successful system login. The functionality of the “Open landing page and browse App website” use case is extended by some other supplemental use cases, and they communicate with the server application. The behavioral diagrams in the subsequent discussion describe the implementation of these use cases from different aspects.

The land suitability index, LS, is computed based on three thematic indices: fertility quality index, FQI; chemical quality index, CQI; and physical quality index, PQI, from Eq. (2):

2 $${\text{LS}}=\mathrm{FQI }\times \mathrm{CQI }\times {\text{PQI}}$$

The selection of parameters that affect each index is based on the growth requirement of the crop. Each index is calculated by multiplying factor scores for each affecting parameter and then raising the product to a power equal to the reciprocal of the number of factors. The numerical rating of the parameter factor scores is based on experts’ suggestions and a review of the literature. The FQI, CQI, and PQI are computed from Eqs. (3)–(5), and as mentioned before, some of the parameters involved in the computation of each index can be omitted and the equation modified accordingly. Based on the value of LS, the land suitability class is determined according to the used suitability rating classification to unclassified, highly suitable, moderately suitable, marginally suitable, and unsuitable.

3 $${\text{FQI}}={\left({S}_{{\text{N}}}\times {S}_{{\text{P}}}\times {S}_{{\text{K}}}\times {S}_{{\text{zn}}}{\times S}_{{\text{OM}}}\right)}^\frac{1}{5}$$

4 $${\text{CQI}}={\left({S}_{{\text{S}}}\times {S}_{{\text{ESP}}}\times {S}_{{\text{CaCo}}3}\times {S}_{{\text{PH}}}\right)}^\frac{1}{4}$$

5 $${\text{PQI}}={\left({S}_{{\text{R}}}\times {S}_{{\text{T}}}\times {S}_{{\text{D}}}\times {S}_{{\text{F}}}{\times S}_{{\text{Y}}}{\times S}_{{\text{HP}}}{\times S}_{{\text{G}}}{\times S}_{{\text{WHC}}}\right)}^\frac{1}{8}$$

**Fig. 11:**
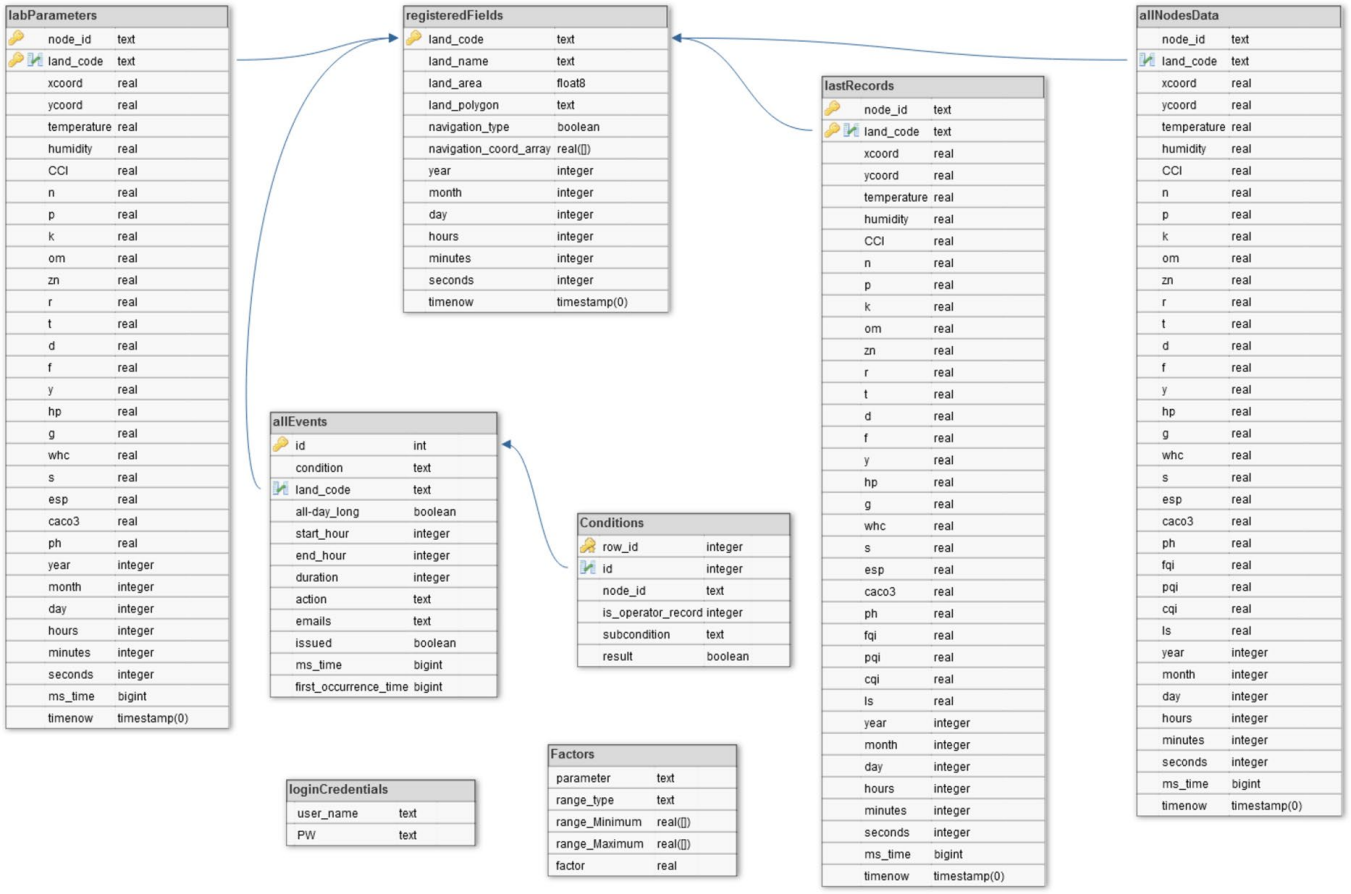
The proposed system database schema

Before delving deeper into describing the interactive behavior of the system, it is worth introducing the stored data, the tables, and relational diagrams; Fig. 11 shows the system database schema. Each of the three tables allNodesData, labParameters, and lastRecords contains the values of the field phenomena: allNodesData stores all the records of real-time values that come directly from the field corresponding to all the nodes deployed in any registered land, and in addition to that, the table record stores the soil quality indices computed in terms of its fields’ values. The table labParameters contains the values of the phenomena determined by on-site measurement or laboratory analysis of soil samples corresponding to all the nodes deployed in all the registered land, and these values are inputted into the system manually. The table lastRecords stores only the last record of each node of its real-time, lab, and computed parameters. The registeredFields table stores the registered land information. The two tables allEvents and Conditions are related to storing the tested event specifications. The Factors table is dedicated to storing numerical rating of factor scores to facilitate changing this rating according to the crop and the suitability assessment purpose. The rating scheme can be expressed as follows: for parameter *x*, the factor score is s1 if *x* < low bound, s2 if *x* $\in$ [low bound, intermediate limit], s3 if *x* $\in$ [intermediate limit, high bound], and s4 if *x* > high bound, where the bounds of these ranges are numerical values. In another possibility, in which *x* takes a discrete value represents a code corresponding to a textual description of *x*, for example, the soil texture can be SiC, silty clay; SiCL, silty clay loam; and/or CL, clay loam, expressed as numerical codes of 1, 2, and 3, and in this case, the range is an array of numerical codes that describe the parameter value.

The Factors table stores the scheme through fields that identify the parameter, the range type whether numerical range or array of discrete values, and fields that identify the bounds of a numerical range, while the codes array is stored in the range_Minimum field, and field for storing the factor score. Data dictionaries for additional explanation of database schema tables are available in the Appendix.

**Fig. 12:**
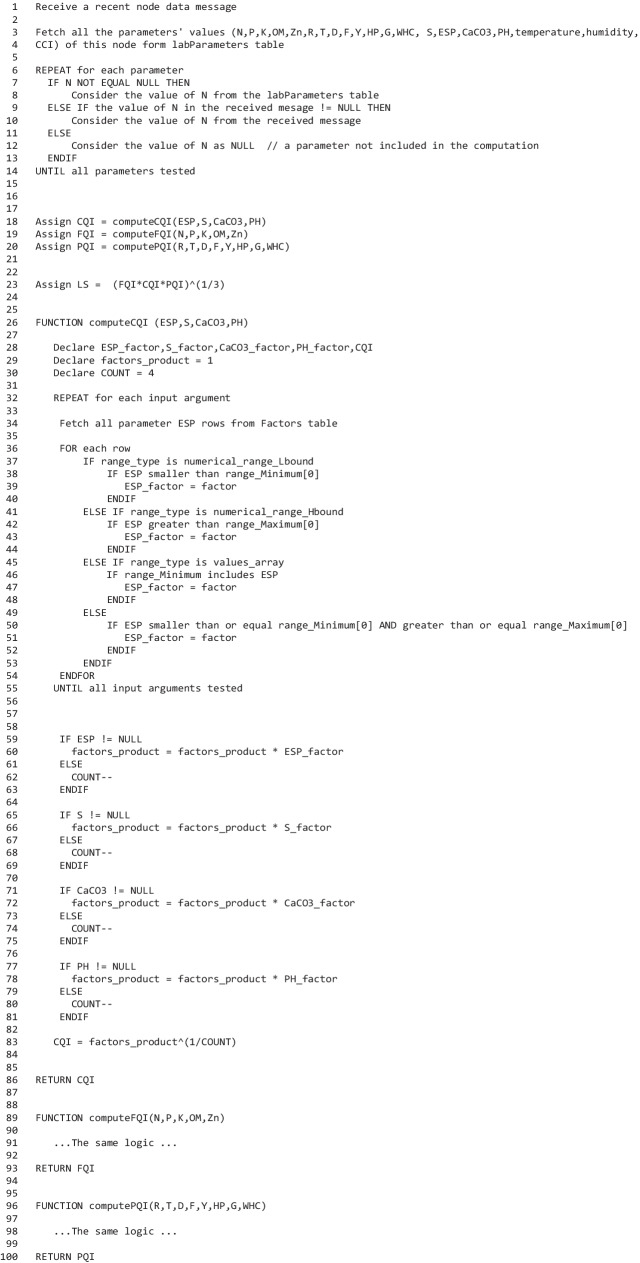
Pseudocode for land quality indices computation

The pseudocode in Fig. 12 represents the computation of the three quality indices. The computation of the indices begins when a data message is received from a node by fetching the values of all parameters of this node from the table that is dedicated to storing the laboratory values. The considered value of each parameter is its fetched value if it is not NULL; otherwise, it takes its value from the received massage whether a numerical value or NULL. The pseudocode then illustrates the computation of the CQI as an example of an index computation. The code loops through the parameters influencing CQI and uses the Factors table to determine these parameters’ factor scores. The If statement in line 37 checks the range type whether it is a numerical range of the low bound only, a numerical range of the high bound only, a codes array, or a numerical range of two limits, and then, it makes the appropriate test to see if the parameter value falls within the range. Then, it considers the factor scores of the parameters in the computation only if it is not NULL and modifies the exponent accordingly.

Figures 13, 14, 15, 16, 17, 18, 19, 20, 21, and 22 represent the message sequence charts of the system services. All the different server environments that run on a single physical server are represented in the charts as a single object “server-side,” and the dynamic webpage generation interactions are ignored in the charts.

**Fig. 13:**
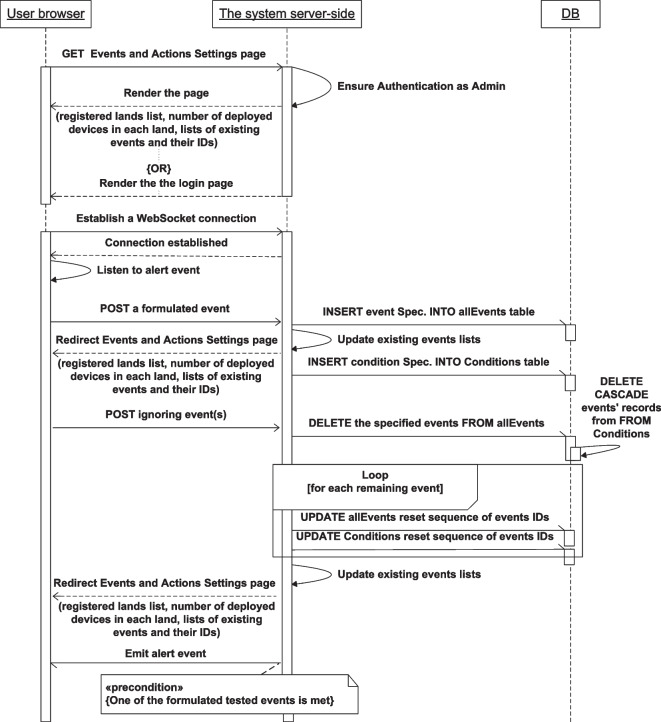
Events and Actions settings service message sequence chart

**Fig. 14:**
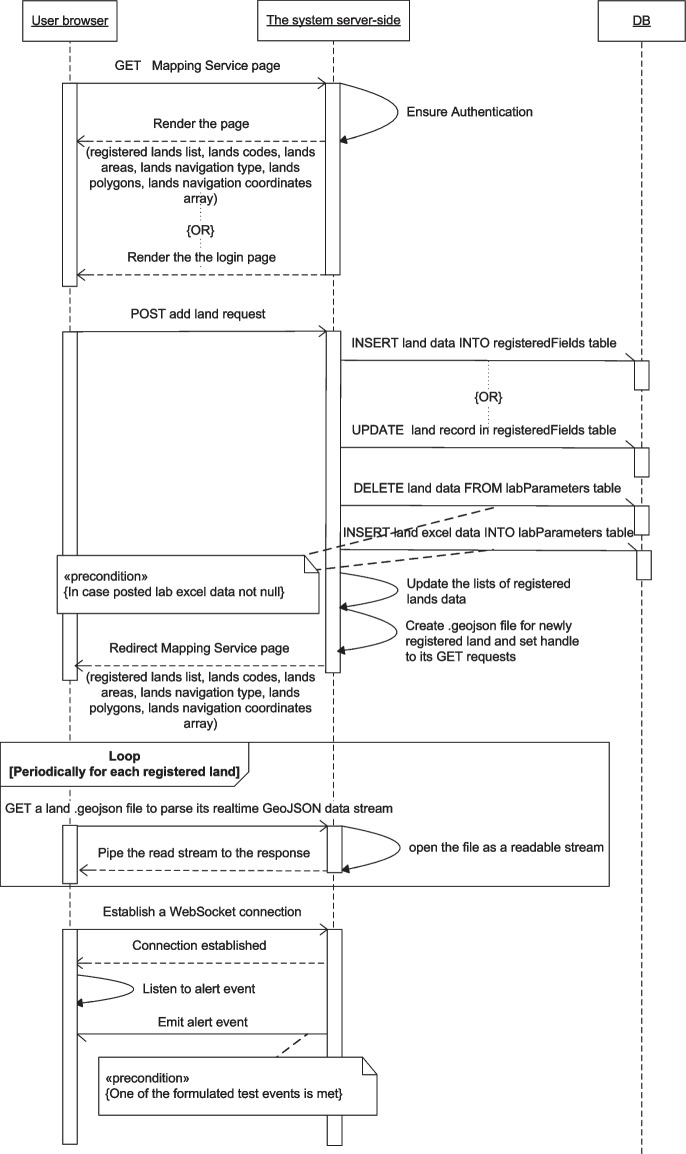
Mapping service message sequence chart

**Fig. 15:**
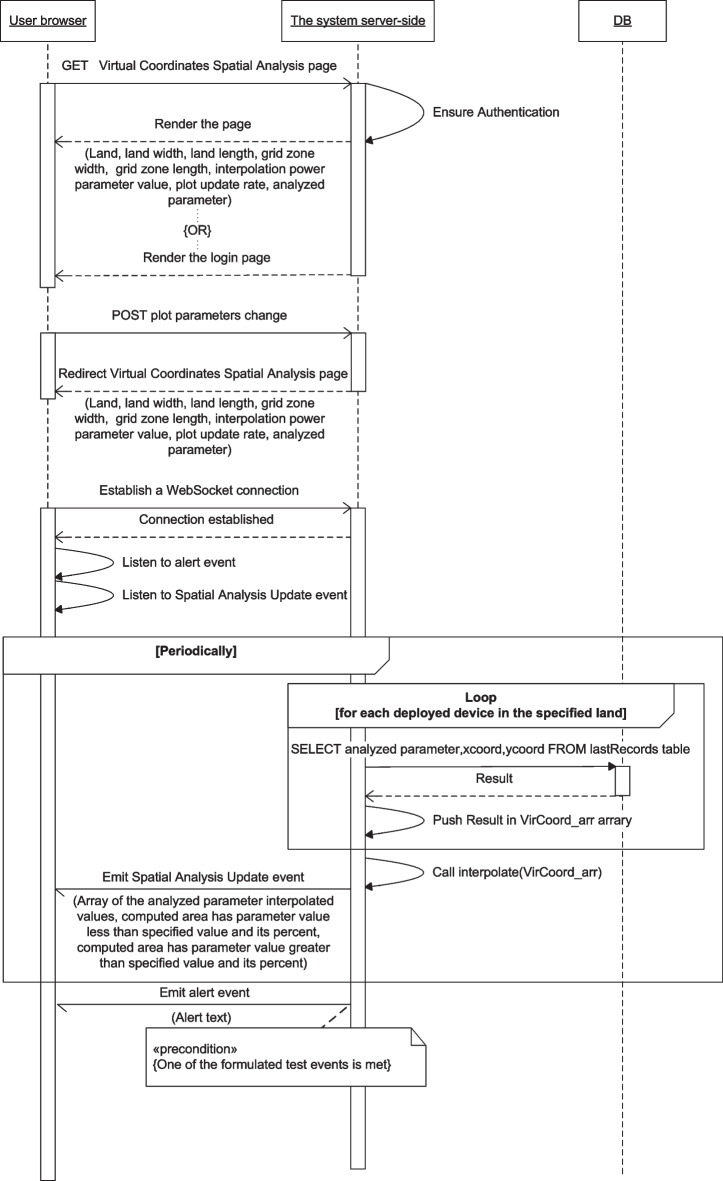
Virtual Coordinates Spatial Analysis service message sequence chart

**Fig. 16:**
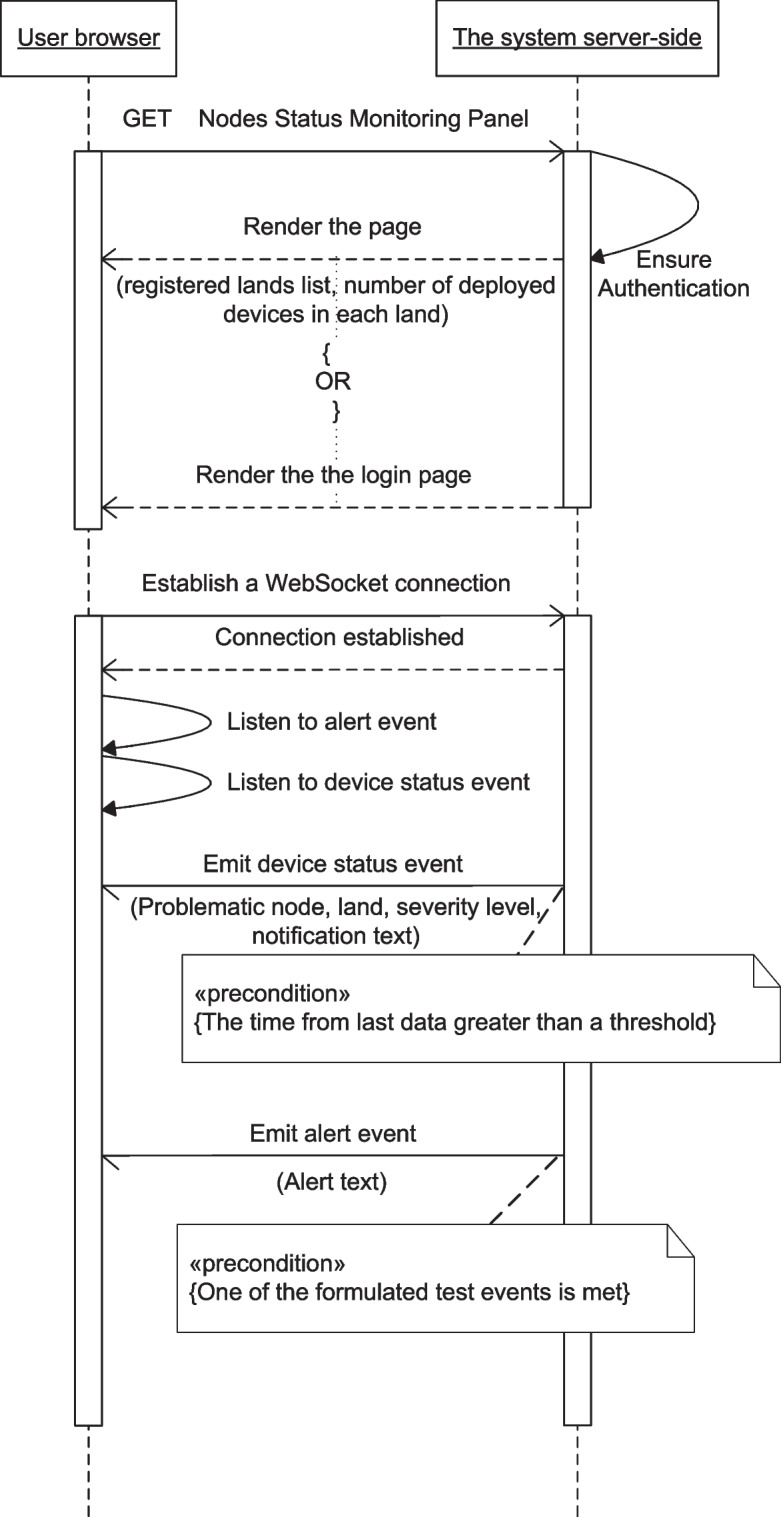
Nodes Status Monitoring service message sequence chart

**Fig. 17:**
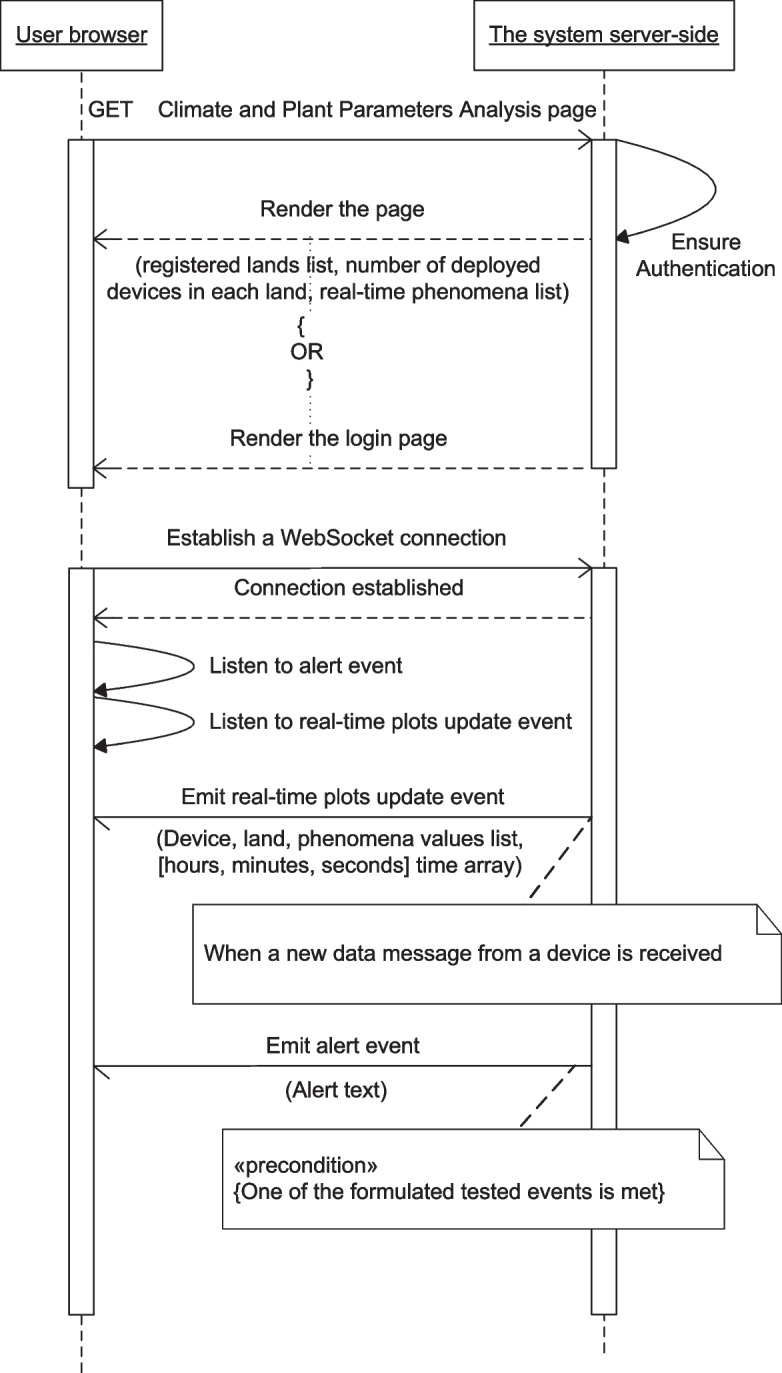
Climate and Plant Parameters Analysis service message sequence chart

**Fig. 18:**
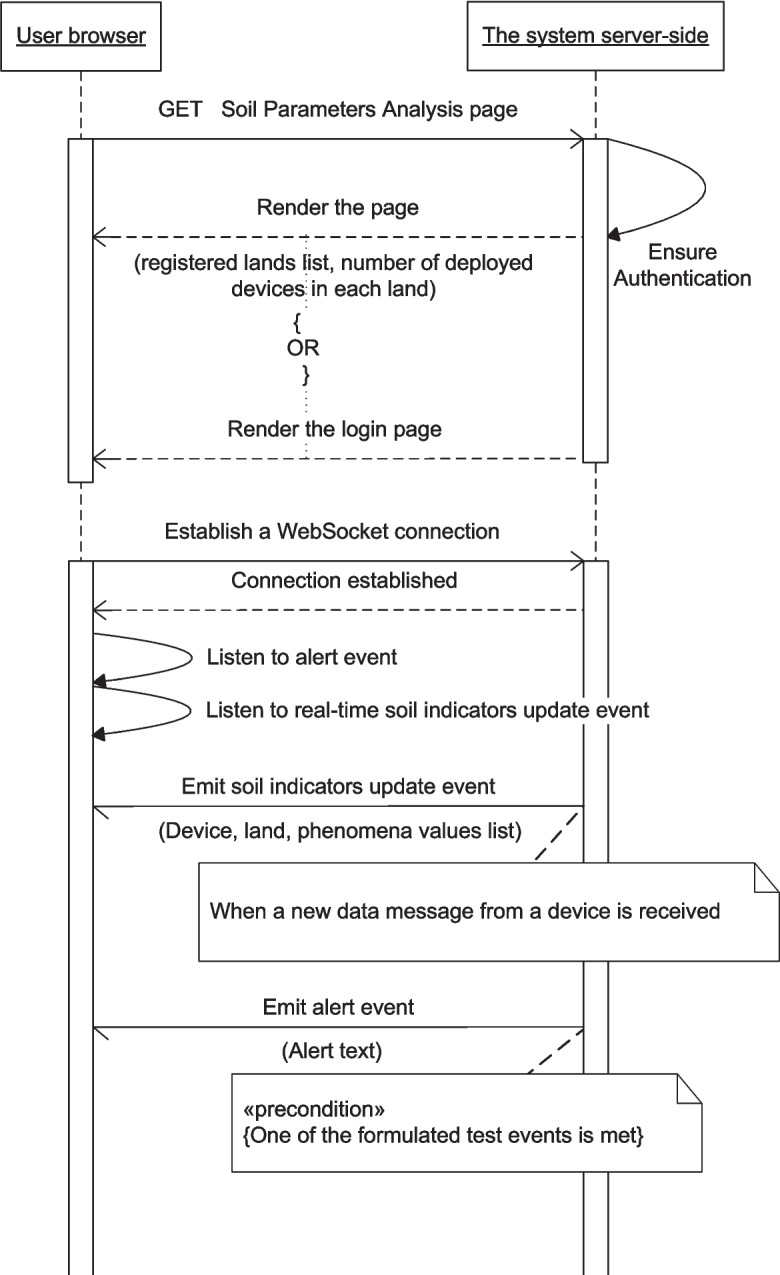
Soil Parameters Analysis service message sequence chart

**Fig. 19:**
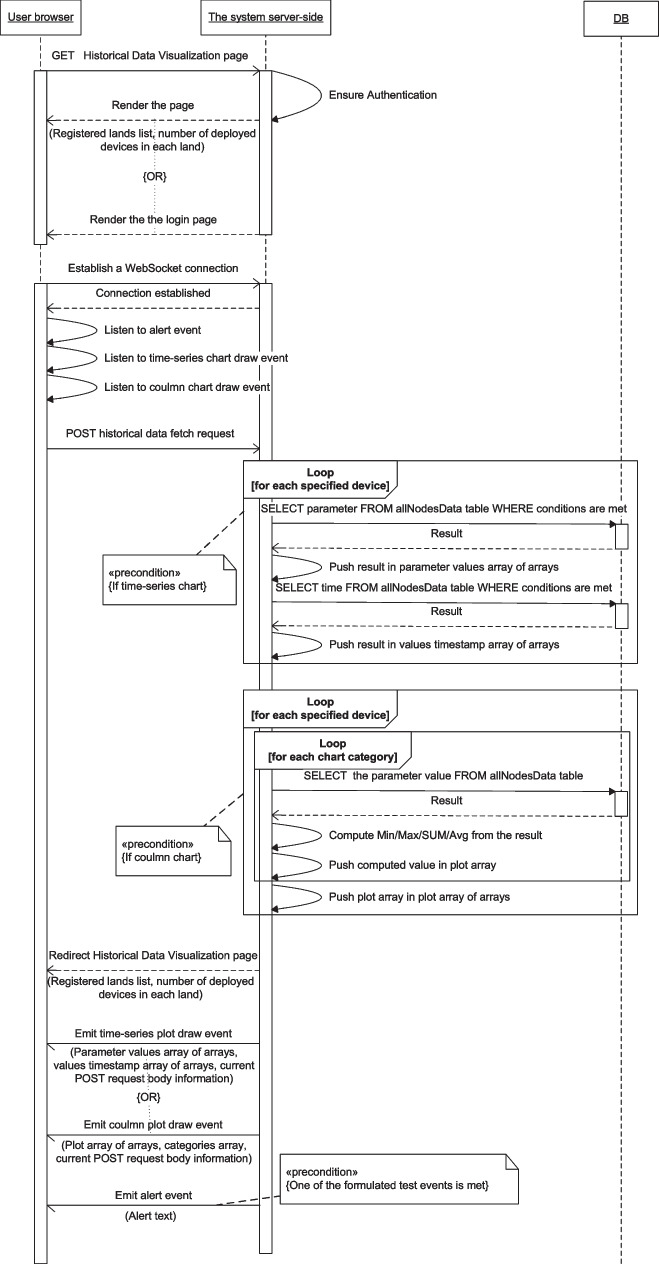
Historical Data Visualization service message sequence chart

**Fig. 20:**
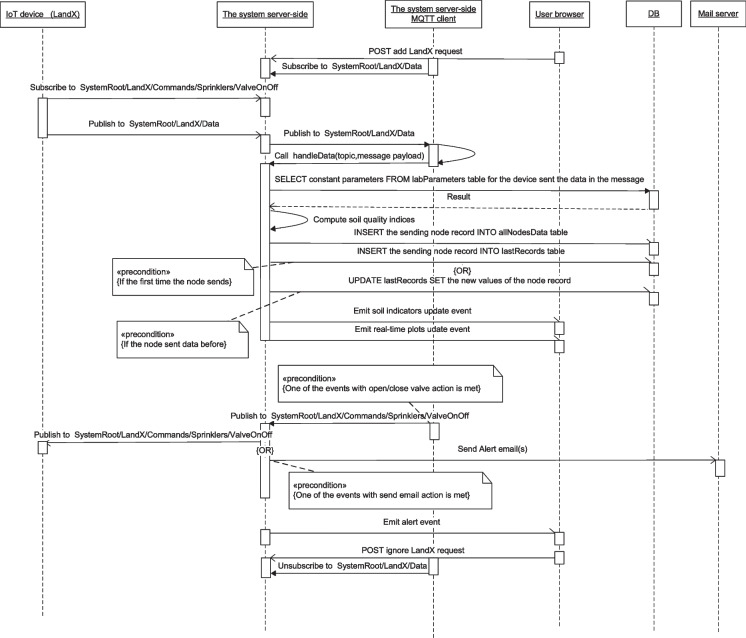
IoT massaging message sequence chart

**Fig. 21:**
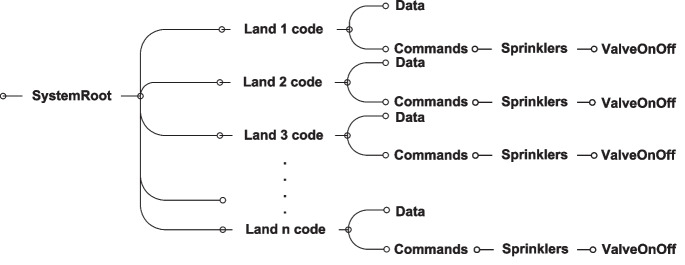
IoT massaging topic tree

**Fig. 22:**
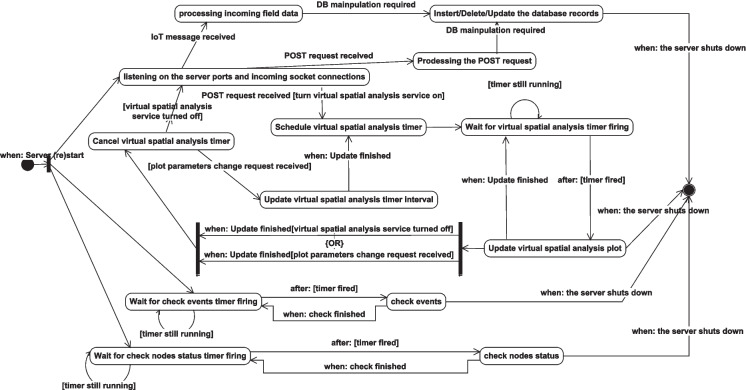
State transition diagram for the proposed system

The Events and Actions settings service includes transactions among three objects shown in Fig. 12. They are the user browser which controls the front-end content of the system, the server-side, and the database. The browser or the front-end code sends the HTTP GET request to the server to retrieve the Events and Actions settings webpage and sends HTTP POST requests with the data of a newly formulated event or the data of existing event(s) to be ignored. The server-side responds to these requests by doing the required interactions with the database to add or ignore the specified events and by rendering and sending the requested page with the appropriate local variables or sending the login page if the authentication failed. All the front-end pages use an established WebSocket connection between the sever and the browser to receive the Alert event.

The Mapping service transactions which are described in Fig. 14 include POST requests to add and ignore lands and the associated database manipulation statements. The server-side continuously updates the GeoJSON representation of the lands’ data, and the Mapping page periodically gets this data to display it on the map.

In the Virtual Coordinates Spatial Analysis service, Fig. 15, the WebSocket connection is used for listening to the Spatial Analysis Update event issued by the server periodically to send the updated data of the parameter considered for the analysis. The browser can post new settings of the 3D plot to be considered by the server when computing the subsequent plot parameter data.

The sequence charts of the three services aggregated to the Real-time Data Visualization service are shown in Figs. 16, 17, and 18. They mainly depend on event-based transactions between the browser and the server.

The Historical Data Visualization as shown in Fig. 19 includes a POST request for historical data retrieval from the database containing the primitives for fetching and preparing the data, upon which the server follows these primitives and submits the result back to the browser through event-based communication.

The IoT massaging–related interactions and topic tree are shown in Figs. 20 and 21, respectively. The MQTT client of the server-side subscribes to the data channels of all the registered lands, while the IoT devices in each land publish data through the land data channel. On the contrary, IoT devices play the role of subscribers but to the channels of the application commands, while the server plays the role of publisher that publishes its commands to the devices through these channels. These interactions entail other transactions with the database, the browser, and the mail server.

The different states and state transitions accompanied by the system can be summarized by the server-side lifetime conditions as depicted in Fig. 22. The server always is in the listening state on the system communication ports, and in the event of receiving a request through them, it transits to the processing state of the received request. Three timers control the server operations, two always-on timers: one of them is for periodically checking all the deployed node status and the other one is for periodically checking the saved events to detect if one of them is realized. The third timer is for periodically updating the virtual coordinates spatial analysis plot, and it is always on only if this service is enabled by a POST request. All the states have a transition to the final state in the event of a server shutdown, but it is not indicated completely in the figure for clarity and simplicity.

**Fig. 23:**
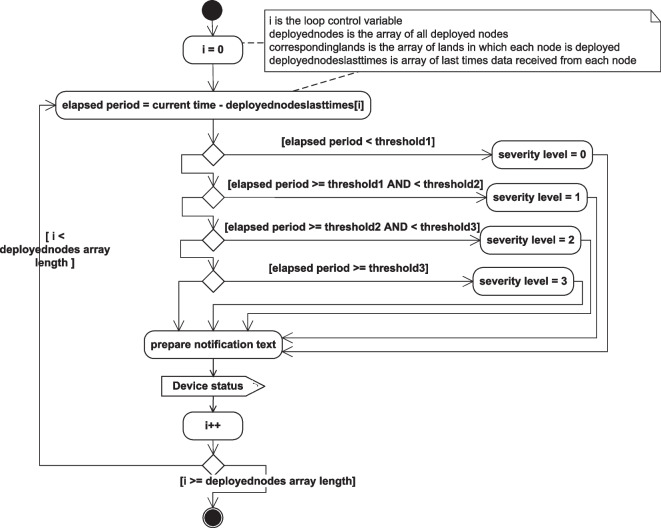
Node status checking activity diagram

**Fig. 24:**
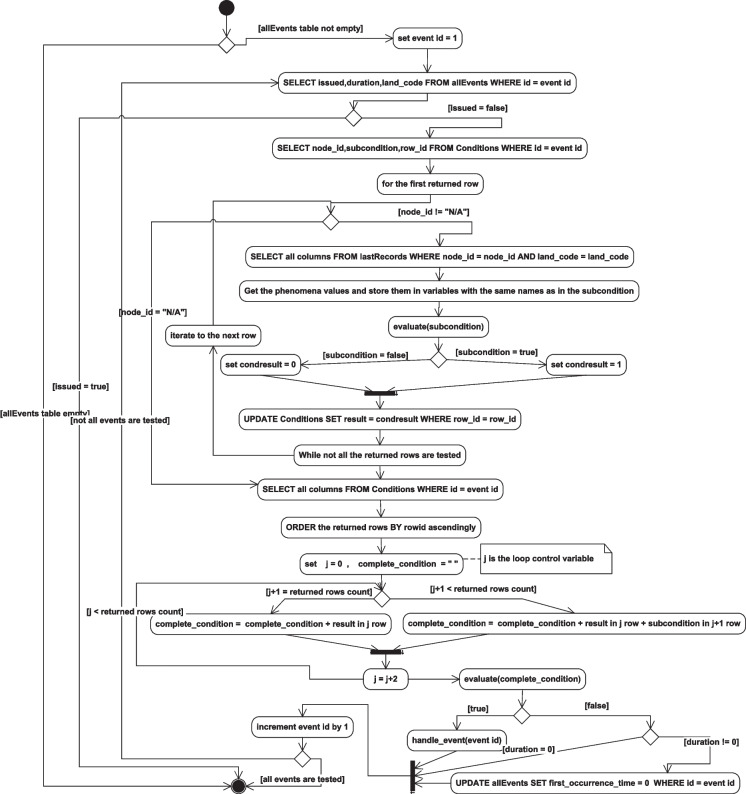
Event checking activity diagram

**Fig. 25:**
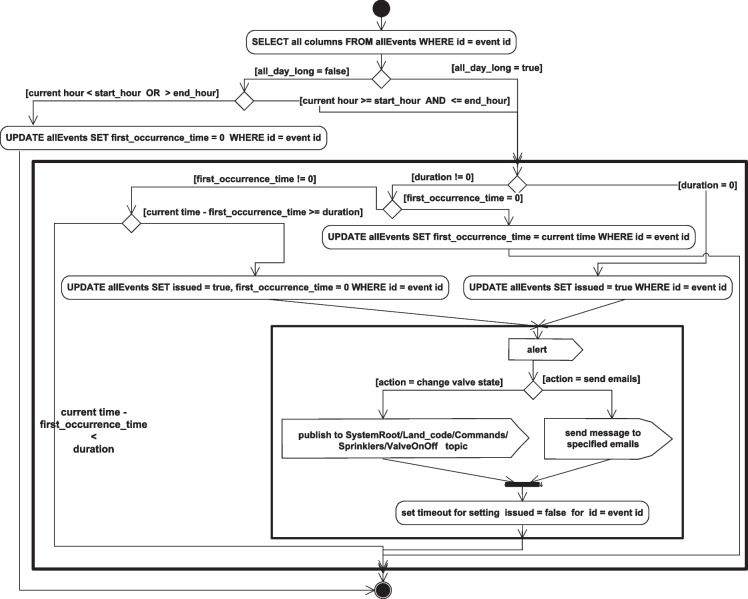
The activity diagram describing the handle_event (event id) function implementation

The flow of some of the system operations and the system’s different function implementation details are described in the following activity diagrams. Figure 23 describes the node status check flow of operations, while Fig. 24 describes the implementation of the event checking activities, and the details of the handle_event(event id) function are further described in Fig. 25.

## Results

This section contains the results of validating the selected technologies and the system functionalities with respect to its IoT use case specification through a small-scale implementation. The validated functionalities are developing a functional Node.js back end, developing an interactive front-end webpage that sends the user inputs, issuing events to the back end, and receiving issued events from the back end, and a functional PostgreSQL implementation can be used to store and retrieve data, an IoT publish/subscribe valid connection from an Internet IoT device to the server and the display of its data in real time, and finally, the spatial analysis functions of the digital map.

### IoT real-time connection validation

**Fig. 26:**
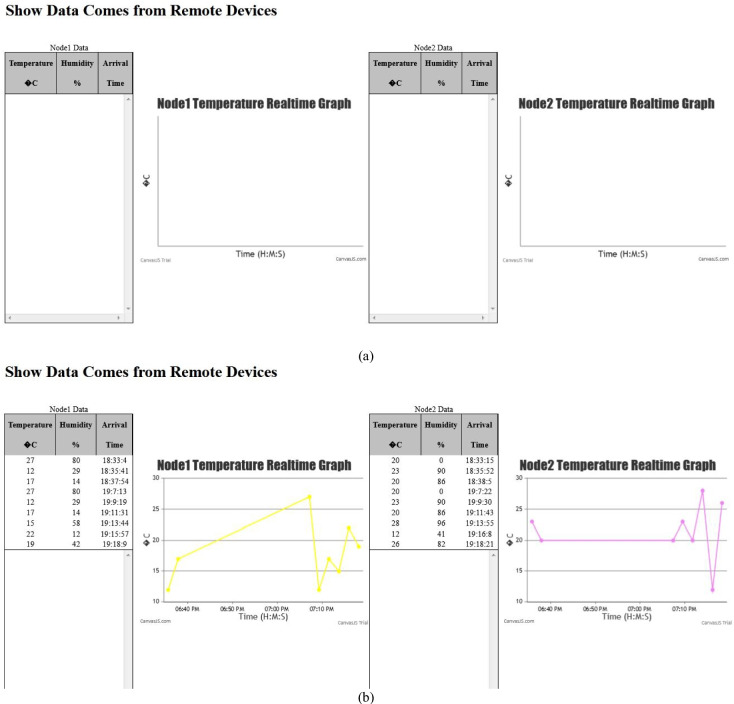
The interactive webpage to test real-time data display **a** before the nodes started to send data, **b** after the nodes started to send data

An interactive webpage is developed to display the data sent by remote devices immediately as it arrives in tabular and graph forms (Fig. 26a). The webpage uses HTML, CSS, JavaScript, and the EJS templating language to embed JavaScript into HTML templates to implement the dynamic table from the list of items sent by the server to the webpage when rendering it and the CanvasJS Charting Library to implement the dynamic graphs. The MQTT library is used for implementing the publish/subscribe interaction, and the Socket.io library is used to enable the real-time web app service for updating the graphs. The Eclipse Paho client library is used for implementing a remote device.

In this validation example, the “mqtt://broker.hivemq.com” public broker is used, where the remote devices Node1 and Node2 are connected to it and publish their data to the topics “Node1/data” and “Node2/data,” respectively. As the server subscribes to these two topics, it receives the data sent through them. Figure 26b shows the tables and the graphs as they are updated with Node1 and Node2 data that is immediately sent by the server via issuing UpdateGraph events.

**Fig. 27:**
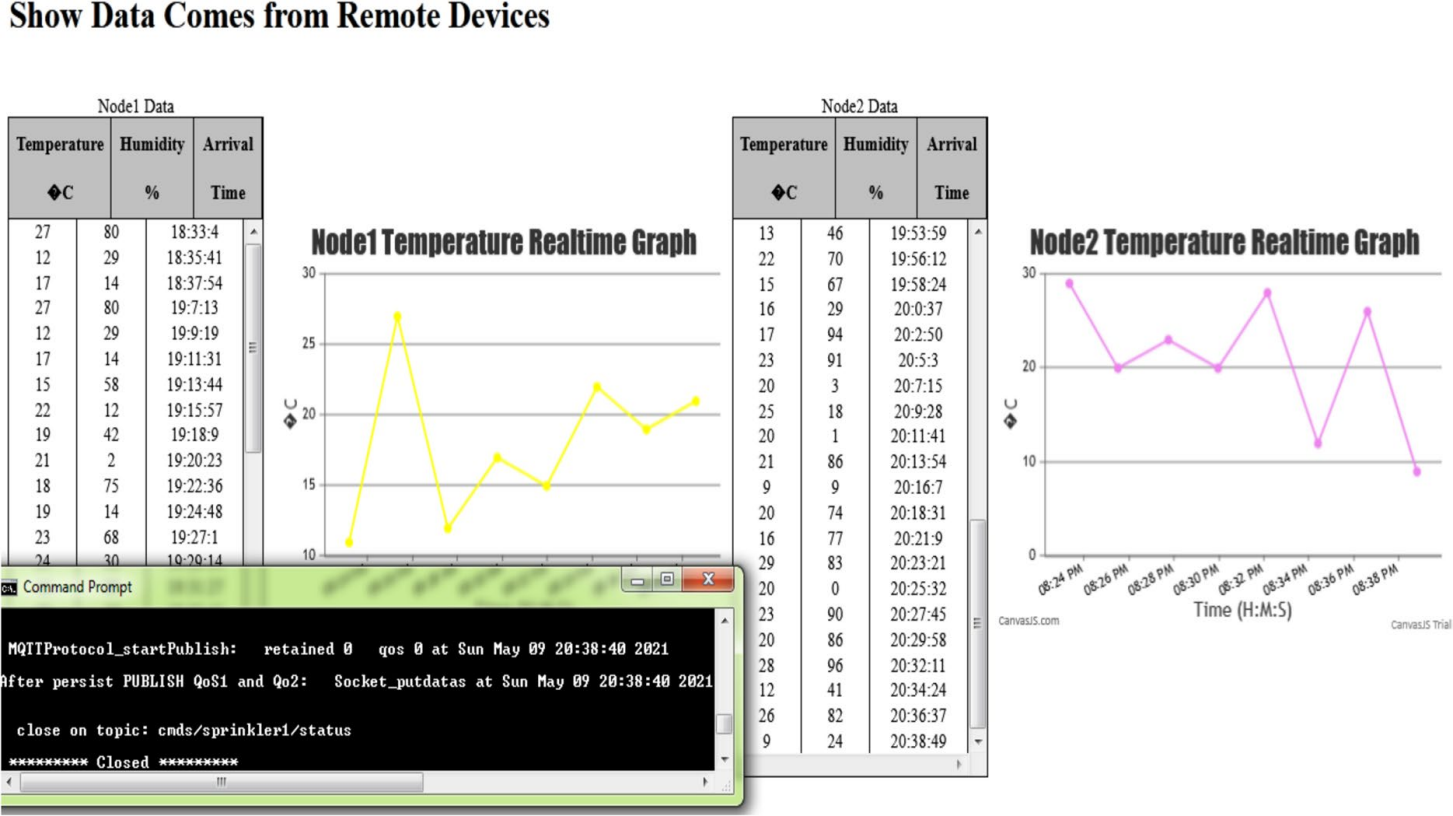
The bidirectional IoT connection test

Node2 is subscribed to the topic “cmds/sprinkler1/status,” the server publishes to this topic a payload “Open” to command the node to open the sprinkler if the node temperature value becomes greater than 20 and the sprinkler is currently closed, and in a similar way, it publishes to this topic a payload “Close” to command the node to close the sprinkler if the node temperature value becomes less than 10 and the sprinkler is currently opened. Figure 27 shows that when the temperature of Node2 fell below 10 and the sprinkler was in the Open status, the server sent the Close commend which was successfully received by the node as indicated by the message “*****Closed *****.”

### Form input and database retrieval validation

**Fig. 28:**
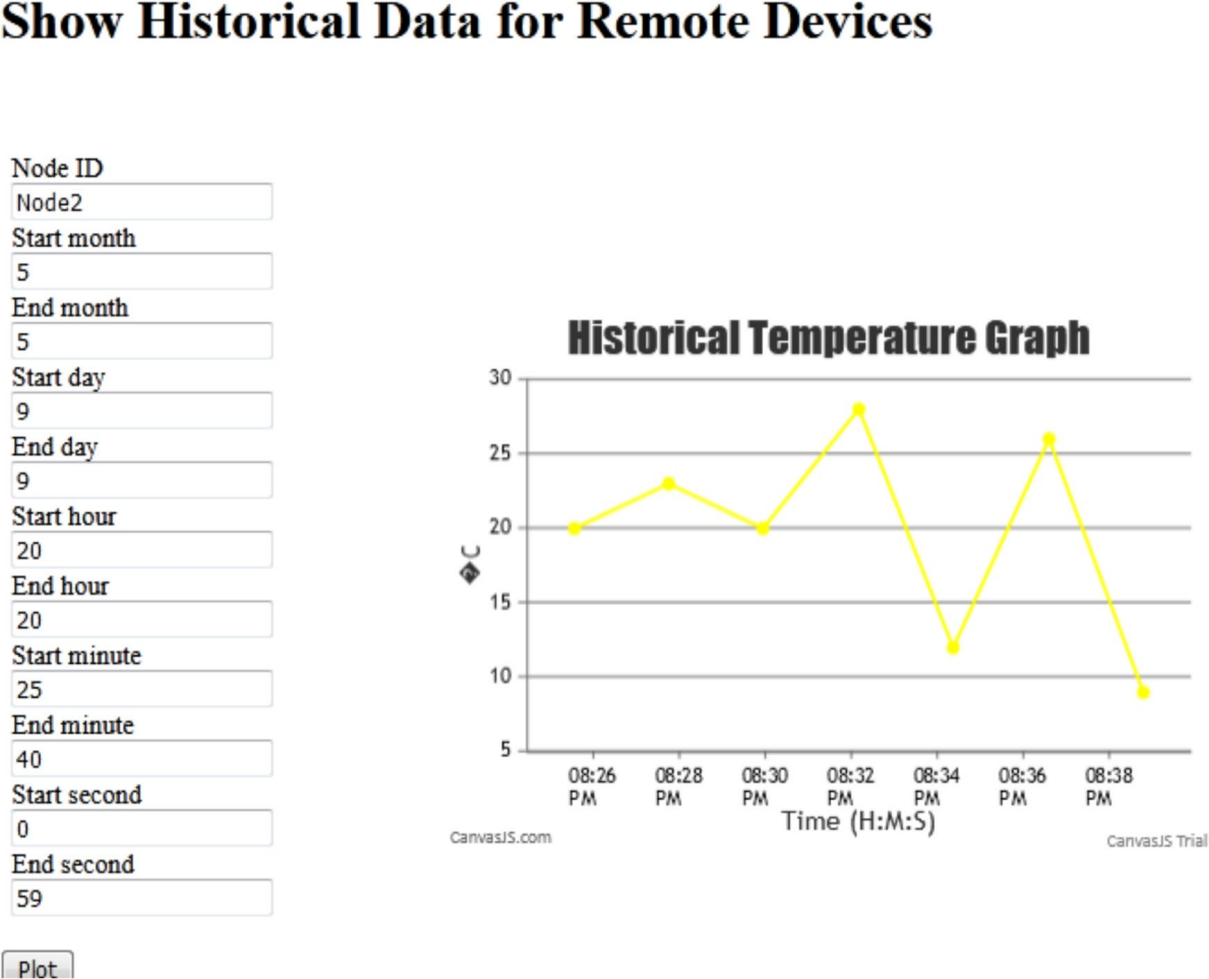
Webpage for validating storage and conditional retrieval of IoT device data from the database

A database table was constructed to store the data of the IoT devices. A webpage (Fig. 28) was developed to submit the specification of the data that the user wants to retrieve from the table. The server fetches the required data and emits an event with it to the webpage where it is displayed in the graph.

### Spatial analysis functionality validation

**Fig. 29:**
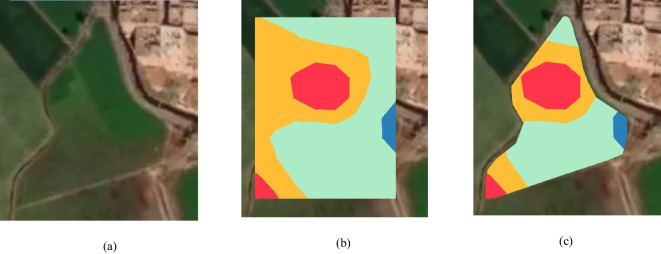
The spatial analysis result using Mapbox and turf library. **a** The study area, **b** the computed isoband fit the Bounding Box of the study region, **c** the isoband fit the area of the study region

Mapbox and turf library were employed to perform the spatial analysis. Two methods were implemented: in the first one, the required phenomenon values of sample data points from a study region (the region appears in Fig. 29a) and the “turf.interpolate” were used to generate a grid of values covering the whole study region. Then, the number of zones and breaks/ranges for the zones values were determined to be four zones with breaks [8,10,16,20,30], and the “turf.isobands” was used to draw the isobands that represent the different zones (the different specified ranges of the interpolated grid points’ values) as shown in Fig. 29b. Then, the isobands were cut to fit the area of the study region as shown in Fig. 29c, where the Mapbox Draw tools were used to delineate the study region boundary.

**Fig. 30:**
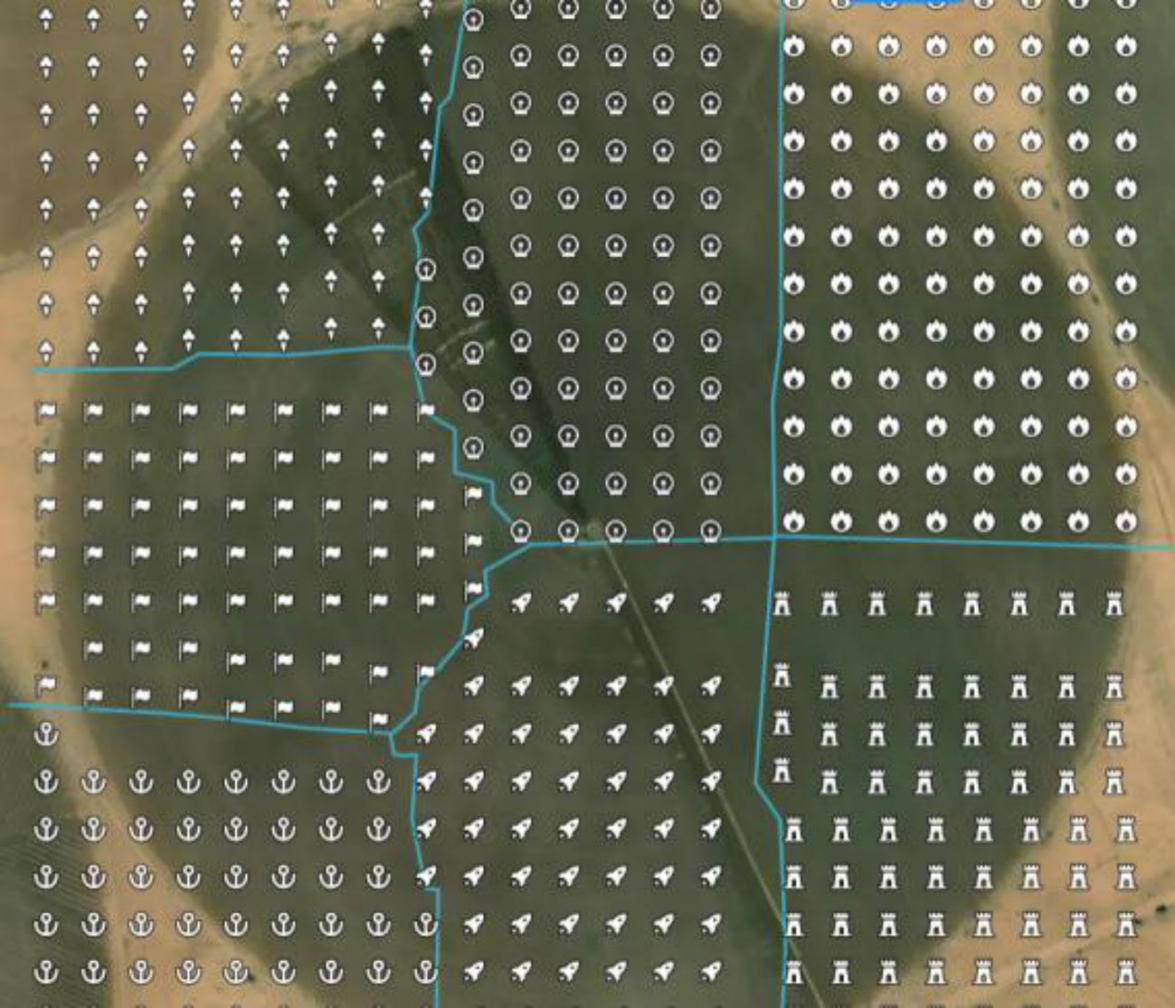
Circular area data clustered into seven clusters using turf.clustersKmeans

In another method, the spatial analysis can be performed using the *K*-means algorithm using turf.clustersKmeans on the interpolated grid values of sample data points. Figure 30 represents another area shape of a study region; here, the numberOfClusters was specified to be seven. As shown in Fig. 30, the algorithm clustered the data into seven clusters:

1. Cluster0: min value 10.015541544598735, max value 26.986562988768455
2. Cluster1: min value 13.929786699318, max value 21.700365700682692
3. Cluster2: min value 12.00532235608656, max value 21.999103602945702
4. Cluster3: min value 13.321658147218638, max value 28.37229042462814
5. Cluster4: min value 15.00167606459235, max value 19.992208792100453
6. Cluster5: min value 12.01204456104, max value 28.992674004
7. Cluster6: min value 13.8663774958, max value 17.238501579

## Discussion

**Fig. 31:**
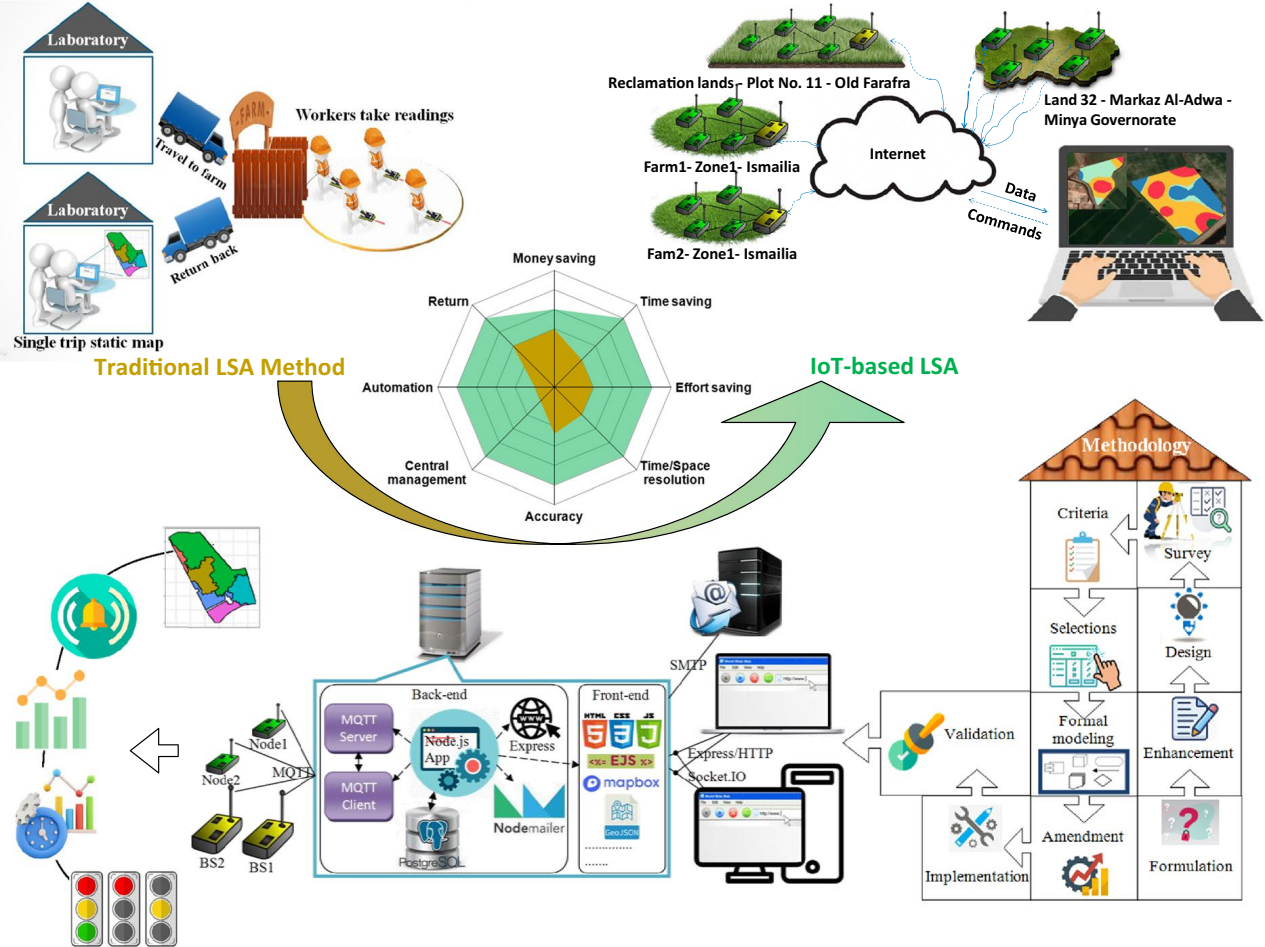
Graphical abstract of the proposed solution

Generally speaking, this paper is about a software solution acting as a part of a complete end-to-end software and hardware system for a farm enterprise. Figure 31 summarizes the work done in this paper in graphical form.

Technology-wise, the solution represents an IoT Web App; application-wise, the solution is mainly a land suitability assessment tool for environmental and agricultural production sustainability; market-wise, it can be considered an enterprise-scale field monitoring and mapping software product.

The IoT use case is formulated taking all of these three aspects into account. Starting application-wise, we turned to the agricultural experts to find out their recommendations on a specific important agricultural aspect that needs to be digitally transformed and performed smarter, and the choice fell on the land suitability assessment process.

**Fig. 32:**
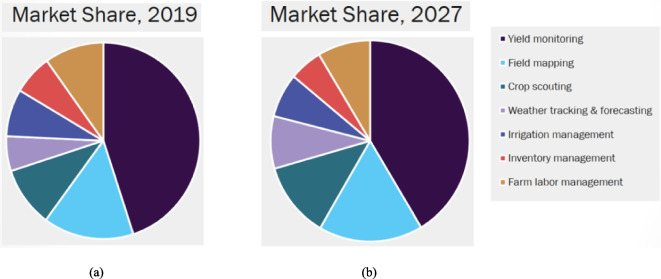
Precision farming market, application revenue movement analysis, 2019 & 2027 (Grand View Research, 2020), CAGR – -% from 2020 to 2027, revenue estimated at USD – Million in 2019. **a** The market share in 2019, **b** the market share in 2027

**Fig. 33:**
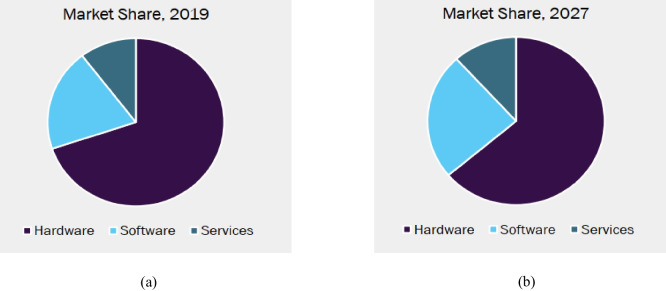
Precision farming market, offering revenue movement analysis, 2019 &2027 (Grand View Research, 2020), CAGR – -% from 2020 to 2027, revenue estimated at USD – Million in 2019. **a** The market share in 2019, **b** the market share in 2027

As a product, estimating its market size helps in identifying whether such a system really has potential customers and whether it is expected for it to succeed and sustain. The precision agriculture market is segmented into seven application types; as shown in Fig. 32, the yield monitoring and field mapping applications are on and expected to remain on the throne of revenue generation. Likewise, as shown in Fig. 33, the market share of software occupies the second rank in the precision agriculture market offerings, and it is expected to increase.

As previously mentioned, the system’s potential customers are owners of large private farms, government for large-area state-owned agricultural lands, and scientists in the field of agricultural and soil applications.

These customers need to take precision decisions on land use and management, decrease field trips, save time and effort, decrease labor costs, prevent the waste of agricultural inputs, monitor the field with a high spatial–temporal resolution, decrease human errors in measurements and analysis, model agricultural operations based on experts’ knowledge, control their farm equipment and conditions remotely, improve crop quality and size, increase profits, preserve the environment, etc. At this time, the system use case can be narrated.

The features of the proposed design are selected to meet these needs and add value in a way that increases the chance of the system to become effective and to gain a competitive advantage. The system is designed not to depend on the human factor in taking readings and analysis; it is prepared to communicate with the smart devices in the field and take readings from them directly, store the data, and process it based on the experts’ models of spatial analysis. In addition to that, it also allows for data input from humans. The spatial analysis and land suitability assessment maps are produced automatically in real time and from historical data. Based on these maps, the users can compare lands and differentiate the application of agricultural inputs to different regions of the land according to their real need. Regardless of the main purpose of the system, its proposed design augments it with other features that generally meet the need and satisfy the customers of the market segments related to the system, such as remote control, detections and alarms, and spatial analysis using virtual coordinates.

A good feature of the proposed system is its ability to interoperate with governmental initiatives and regional/international spatial data infrastructures. Every country has a land cadastre database, which stores cadastral information and metadata for its agricultural lands such as unique identification, owner information, area, location, boundaries, soil quality characteristics, irrigation system, and crops. This information can be used by interested governmental or non-governmental entities, with appropriate authorization privileges, to improve the land-use analysis and thus achieve sustainable land management.

Recently, many countries, including Egypt, have turned to digital transformation and developed strategies for it, including, as an important part, the automation of the land cadastre database. Egypt is working to automate the agricultural tenure system and build a comprehensive and integrated geographic database on agricultural tenure, land cadastre, and related activities at the republic level, which will lead to the integration of geographic data for government agencies and the ease of exchanging, displaying and analyzing information and preparing future plans through establishing a national center for spatial data. The establishment of this center would ensure the provision and availability of a base map for all sectors of the state, with the aim of raising the efficiency of planning decisions, providing cooperation and coordination in implementing policies and foundations of uses, and facilitating the sharing and exchange of information between government agencies.

Assuming that the local land cadastre database follows the international standard (Land Administration Domain Model (LADM)) in its implementation, the National Center for Spatial Data is the source of the data related to the two packages: Party and Administrative. The proposed system is related to the Spatial Unit package and its sub-package Representation and Survey, and in addition, the model can be extended by associating classes related to land suitability indices. The system will be the source of the spatial and soil-related data. The connection of the system to the local land cadastre database can be through a remote database connection or through an MQTT connection. The second option is preferred for several reasons, such as bandwidth, security, and loose coupling, and can be implemented by integrating the local land cadastre database with an MQTT client.

The system topic tree will be expanded to include a topic, under the land code level, related to land suitability indices. The MQTT client of the database system subscribes to this topic for all the registered lands in the proposed system, while the MQTT client in the proposed system back end publishes this topic periodically for all lands. The published data can be the land suitability assessment map itself. This requires the database system to securely maintain land codes associated with the corresponding land data and also requires an update in the MQTT server access control list (ACL) for restricting the subscription to these topics to the MQTT client of the local land cadastre database and the publication on each of these topic to the relevant land, where the client ID of each land represents its land code. This is of course, in addition to the existing rules that restrict the subscription/publication between the proposed system back end and the MQTT clients of the lands.

Through the standard format that the local database follows, external databases can connect to it and access its stored data based on the established database authorization rules.

**Fig. 34:**
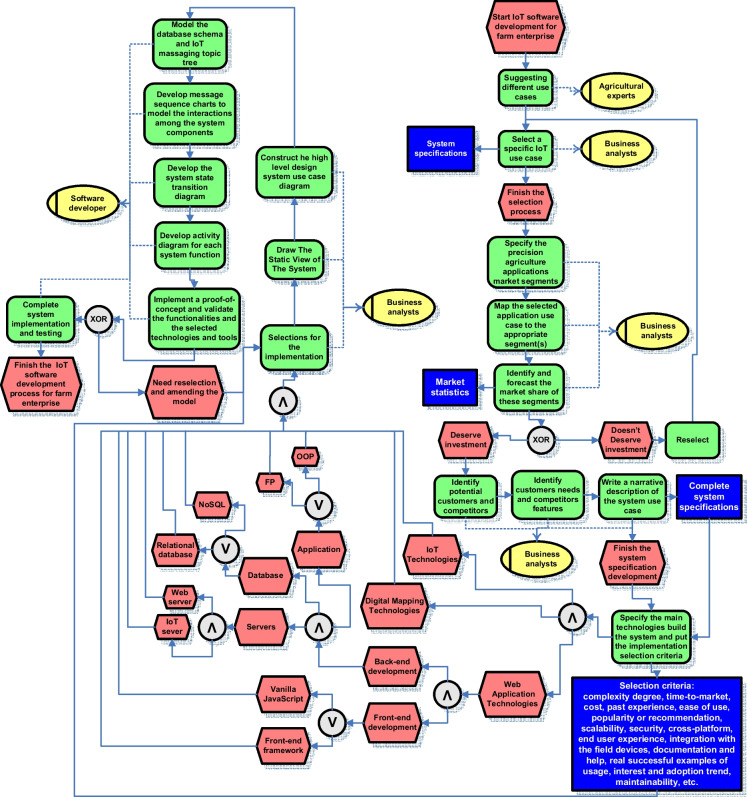
Generalized IoT software development process for farm enterprise

The technology-wise design entails selecting the IoT enabling technologies and tools and before that putting the criteria upon which the selection will be made. The process followed in developing the proposed IoT system can be summarized and generalized as shown in Fig. 34.

Another important matter that cannot be overlooked and is crucial for satisfying the needs of such enterprise-scale IoT system customers is ensuring accurate readings of the IoT devices. Ensuring and improving sensor data quality can be done in several stages and forms. During device design, sensors can be calibrated by comparing the sensor reading to a standard value. The accuracy and precision of readings can be improved through the calibration process by adjusting the design, including the sensor settings, and using appropriate filters to improve the signal-to-noise ratio, until acceptable accuracy is achieved. It is recommended to perform the calibration over the full range of the sensor reading and in different environmental conditions. Also, periodic recalibration is recommended to tackle the drift of sensor readings over time.

Continuous monitoring together with using appropriate validation methods of sensor readings is important to ensure data accuracy and precision. The aim of validation is to detect device malfunction and anomalies in devices’ readings such as bias, dropout, and outliers.

The number of published literature on the topic of designing software systems for land suitability assessment, soil spatial analysis, and management zone delineation is not considered many. Some rely entirely on manual input, and others exploit the embedded system and web technologies to automate and improve some processes done manually in the commercial geospatial processing programs that were not specifically developed for agricultural applications, but they have not exploited the IoT technology. A sample of previous work will be discussed below.

The research by de Freitas Coelho et al., (2018) proposed an embedded system consisting of a single-board computer and sensors and running an application developed using Python and PyQt designed to perform the data analysis immediately after data collection, eliminating the need to come back and forth frequently to and from the laboratory to collect more data from the field for analysis. The application first filters the data through outlier and inlier analysis, then performs kriging interpolation, and finally produces and visualizes the spatial analysis map resulting from *K*-means clustering. This system did not benefit from the communication network technology, as the readings are taken manually and through a single device, which adds effort and time consumption until a single collection of field data is completed and makes it unsuitable for large-area fields. In addition, the motivation of the work which is avoiding the need to return to the field to collect more data to tackle problems in the sampling strategy can be addressed by performing the analysis done by the proposed system using any PC or laptop in the field. Another limitation of the system is the use of collected points in defining the field boundaries.

The research by Werner et al. (2020) developed a web-based spatial analysis application integrated with R language for the statistical and geostatistical analysis and creation of thematic maps. It offers generating the maps using both inverse distance weighting and kriging methods. The application automates some processes such as fitting the best model for a semivariogram. Sample data was used to validate and calibrate the application output with ArcMap. The work describes the application software layer that provides a friendly interface to the user but generates grayscale maps.

A software system for multi-criteria LSA for different types of cultivating crops in tropical and subtropical regions was proposed by Elsheikh et al. (2013). It offers the ability to add a new type of crop by adding a new record to the crop records. It is built using GIS Model Builder, Python scripts, and Visual Basic and uses attribute tables and digital mapping with GIS capabilities to display the output upon which the decision-makers build their decisions. The outputs were validated by subjective comparison based on knowledge of whether the crop was already grown on the land assessed, and expert judgments were also consulted to validate the outputs.

The research by Kang et al. (2012) addressed the use of IoT technologies for zone management delineation. It does not go beyond a conceptual model that specifies the possible architecture and usage of such a system.

Postolache et al. (2023) found that there is no large-scale or commercially available IoT system for horticulture and then proposed an IoT-based system for soil fertility evaluation in horticultural farms to aid in detecting plant stress and improve irrigation and fertilization decision process which needs not only the current value of soil nutrient but the historical nutrient data. The sensing nodes sense some soil and climate parameters and use Wi-Fi or LoRa for communication. The sensed parameters are soil moisture, pH, and N, P, K, relative humidity, and temperature and it offers plug-and-play sensor extension. The system contains a mobile application used to visualize measurement data points, as well as graphs of the time change in parameter value. The sensibility of the sensors and the functionality of the system were tested.

In Martis et al.’s (2022) study, machine learning enabled an IoT platform to predict a crop suitable for the Kharif and Rabi seasons for cultivation. The prediction model was trained with different datasets and used to perform time-invariant classification. The system also provides suggestions to enhance soil fertility. The system is composed of end device sense N, P, K, EC, and pH; a relay unit is used to collect and convey data in a prespecified frame format using MQTT, and the communication between devices is through Wi-Fi and LoRa; and an intelligence unit runs the machine-level algorithms as a service on Dual Intel Xeon. From the prediction results, a rule-based recommendation system was designed to specify the best soil treatment. Table 3 infers specific characteristics of these works compared to the proposed system.

**Table 3:** Comparison between the proposed system and previous work

	The proposed system	de Freitas Coelho et al., (2018)	Werner et al. (2020)	Elsheikh et al. (2013)	Kang et al. (2012)	Postolache et al. (2023)	Martis et al. (2022)
IoT-based	Yes	No	No	No	Yes	Yes	Yes
Technologies survey and selection criteria	Yes	S.W. selection criteria not addressed	Technologies survey not presented	No	No	Technologies survey not presented	No
Reproducible software design with standard visual modeling	Yes	No	No	No	No	No, but explained with relatively more details	No, but the part of machine learning is explained in detail
Support of a lot of sensing modalities	Yes	The BeagleBone Black computer has ports to connect sensors, but specific sensors are not specified	Yes	Yes	May be	Yes	No
LSA support	Yes	No	No	Yes	May be	No	Yes
Spatial analysis and/or management zone delineation support	Yes	Yes	Yes	No	Yes	Yes	Spatial analysis
Flexibility in LSA model implementation	Yes	No	No	Yes	May be	No	Yes
Large-area support	Yes	Not suitable for large area	Moderate	Moderate	Yes	No	No
High spatial and temporal resolution	Yes	High spatial resolution	Moderate	Moderate	Yes	Low spatial resolution	Yes
Preparation of raw data (filtering)	No	Yes	Yes	No	May be	No	Yes
Centralized operation	Yes	No	No	No	Yes	Yes	Yes
Prescription map generation support	Yes	No	No	No	Yes	No	No
Validation and calibration	Selected technologies and implemented functionalities were validated	Functionalities were validated	Yes	Yes	No	Yes	Yes
Any land-shape support	Yes, determined accurately using the powerful tools of the digital mapping toolbox	Yes, but the boundaries determined by collected points, thus may be not highly accurate	Yes	Yes	May be	Yes	Yes
Additional functionalities {climate and plant parameters analysis, climate and plant parameters analysis virtual coordinates spatial analysis, historical data visualization, etc.}	Yes	No	Yes, descriptive statistics of sample data	No	No	Yes	No
Manpower requirement	Low	Relatively high	Relatively high	Relatively high	Low	Low	Low
Cost	Relatively high	Relatively low	Relatively low	Relatively low	Relatively high	Relatively high	Relatively high

## Conclusion and future work

This paper presents the development of a conceptual design of a dynamic system that accommodates the spatial–temporal dynamics of the agricultural soil characteristics to realize a land suitability assessment (LSA) based on the factor analysis method. The IoT-based LSA software system is built using other enabling technologies. It utilizes web development to augment the system with a user-friendly interface that can be opened by any browser from anywhere. The system uses the database technology to store the raw and processed field data. Furthermore, the system uses digital mapping toolboxes to manage the geographic information of the agricultural fields and the data layers of their sensed data. The paper reviewed the recent trends in the system-enabling technologies and the selection of the approaches that were used to design and implement the proposed system.

A vanilla JavaScript front end with some libraries for providing some functionalities communicates with a Node.js back-end through HTTP methods and WebSocket communication. The Node.js application deploys modules for the web server, MQTT client, and server; it communicates with the devices deployed in the field using the MQTT protocol and communicates with the PostgreSQL database.

The device tier of this IoT system contains “class A” LoRaWAN end devices that are deployed according to the soil sampling method used, with a LoRaWAN gateway serving a circular area of at least 18,000 feddan through star-of-stars topology. The node contains a LoRa transceiver and four sensors to measure soil parameters N, P, K, S, WHC, D, PH, T, and F, and furthermore, it contains a GPS module to assign geospatial positioning information to the sensed data. Depending on the sensors integrated into the node, three types of nodes can be produced: the first one contains the soil NPK sensor that senses soil fertility parameters, the second one contains the tilt sensor and acoustic sensor that sense some soil physical properties, and the third one contains the soil nutrient sensor that senses some soil chemical and physical properties. These different types of nodes add the possibility of eliminating the involvement of some parameters in the analysis and reducing the cost of the solution.

Message sequence charts, deployment, use case, activity diagrams, database schema, and topic tree are used to describe the static and dynamic views of the system design. The paper validates only the functionality of the selected technologies with a small-scale implementation example.

Referring to the description of the proposed system, the discussed previous related work, and Table 3, it can be concluded that the proposed system is the only system that benefits from the IoT technology and offers the LSA together with spatial analysis and prescription map generation facilities at large-scale areas. The proposed work is the only work that augmented the system design description with a survey of possible implementation technologies before making selections based on criteria; the design is also augmented by reproducible standard visual modeling. The proposed system offers some additional functionality which can be described as a powerful precision agriculture software and not a dedicated program to perform specific functions. The sketched generalized IoT software development process for farm enterprises is also considered a contribution of this work.

On the other hand, we find that the system has some shortcomings, such as not performing filtration or any preparation of the raw data first before storing and processing it. Regarding the system cost, the IoT technology in general is expensive, but what matters are the benefits resulting from its application and the payback period of each specific use case. The work in this paper is dedicated to the low-level software layer of the system use case and the selected technologies, and implemented functionalities have been validated. Other systems have calibrated the results with real data as they feature a complete system including an implementation of a web or mobile application. The application software layer of the proposed system is addressed in Mohammad EL-Basioni et al.’s (2022) study, where the system output is calibrated with real data. In summary, the work in this paper represents the specification of a low-level software layer of an IoT-based system for a use case not considered before and has advantages over similar previous work.

The proposed design needs enhancements to represent channels for future work. It is better to provide different options for interpolation methods; it is better to provide different options for LSA methods; while maintaining the advantage of being able to set different crop criteria, it is necessary to support more than one crop at the same time; and a complete implementation of this generalized system with large-scale real experiments, accompanied by recording, analyzing, and discussing the results to assess the real impact of the system, needs to be addressed further.

## Data Availability

Not applicable.

## Appendix. This appendix contains data dictionaries for the database schema tables depicted in Fig. 11

Tables 4,5,6,7,8, and 9.

**Table 4:** The data dictionary of allNodesData, a table that stores all readings (real-time and laboratory information) from all devices (sensing points) in all registered lands

Column name	Data type	Constraints	Description
node_id	text		Unique identifier for a sensing device in a specific land
land_code	text	Foreign key, Ref. table: registeredFields, Ref. field: land_code	Secret code specific to each registered land
xcoord	real		latitude coordinates of the device, i.e., the reading, in decimal degrees
ycoord	real		longitude coordinates of the device, i.e., the reading, in decimal degrees
temperature	real		Temperature reading
humidity	real		Relative humidity reading
CCI	real		Chlorophyll content index
n	real		Soil nitrogen
p	real		Soil phosphorus
k	real		Soil potassium
om	real		Soil organic matter content
zn	real		Soil zinc
r	real		Soil drainage
t	real		Soil texture
d	real		Soil depth
f	real		Soil slope
y	real		Soil surface stoniness
hp	real		Soil hard pan depth
g	real		Soil hydraulic conductivity
whc	real		Soil water holding capacity
s	real		Soil salinity
esp	real		Soil exchangeable sodium percentage
caco3	real		Soil carbonate content
ph	real		Soil pH
fqi	real		Soil fertility quality index
pqi	real		Soil physical quality index
cqi	real		Soil chemical quality index
ls	real		land suitability index
year	integer		The year the real-time reading was received
month	integer		The month the real-time reading was received
day	integer		The day the real-time reading was received
hours	integer		The hour the real-time reading was received
minutes	integer		The minute the real-time reading was received
seconds	integer		The second the real-time reading was received
ms_time	bigint		The time the real-time reading was received, in milliseconds, since a reference date and time
timenow	timestamp without time zone		The date and time the real-time reading was received

**Table 5:** The data dictionary of labParameters, a table that stores only all the manually entered readings for field measurements and laboratory analysis at the same sensing points—devices’ locations

Column name	Data type	Constraints	Description
node_id	text	Primary key	Unique identifier for a sensing device in a specific land
land_code	text	Primary key, Foreign key, Ref. table: registeredFields, Ref. field: land_code	Secret code specific to each registered land

The same information of these column names in the other tables

**Table 6:** The data dictionary of lastRecords, a table that stores only the last readings (real-time and laboratory information) from all devices (sensing points) in all registered lands

Column name	Data type	Constraints	Description
node_id	text	Primary key	Unique identifier for a sensing device in a specific land
land_code	text	Primary key, Foreign key, Ref. table: registeredFields, Ref. field: land_code	Secret code specific to each registered land

The same information of these column names in the other tables

**Table 7:** The data dictionary of registeredFields, a table that stores the data of lands registered in the system

Column name	Data type	Constraints	Description
land_code	text	Primary key	Secret code specific to each registered land
land_name	text	Char length: Max. 40 character, not NULL	Appropriate textual description identifies this land
land_area	Double precision		The land area in square meters
land_polygon	text		GeoJSON string encodes the land's circumference polygon
navigation_type	boolean		TRUE means navigate to a certain known point in the land, FALSE means navigate to the bounding box of the area
navigation_coord_array	real[]		Latitude and longitude coordinates: whether two coordinates of a single navigation point or four coordinates of the southwestern and northeastern corners of the landbounding box
year	integer		The year the land was registered or updated some of its information
month	integer		The month the land was registered or updated some of its information
day	integer		The day the land was registered or updated some of its information
hours	integer		The hour the land was registered or updated some of its information
minutes	integer		The minute the land was registered or updated some of its information
seconds	integer		The second the land was registered or updated some of its information
timenow	timestamp without time zone		The date and time the land was registered or updated some of its information

**Table 8:** The data dictionary of allEvents, a table that stores all the formulated events

Column name	Data type	Constraints	Description
id	integer	Primary key	Serial number identifies the event
condition	text		The condition to be tested written in the predetermined format
land_code	text	Foreign key, Ref. table: registeredFields, Ref. field: land_code	Secret code specific to each registered land
All_day_long	boolean		TRUE means test whether the condition is met at any time of the day FALSE means test whether the condition is met at the specified period
start_hour	integer	0–23	In case All_day_long is set to 0, this represents the start hour in the test period
end_hour	integer	0–23	In case All_day_long is set to 0, this represents the end hour in the test period
duration	integer		If not NULL, the event will only be issued if the condition is met and remains fulfilled for this duration in millisecond
action	text		Specification of the action to be taken if an event is issued
emails	text		Comma-separated list of email addresses if the action is to send email message
issued	boolean		Indicates whether an event is issued or not. Used to control the validation of events
ms_time	bigint		The time an event is inserted, in milliseconds, since a reference date and time
first_occurrence_time	bigint		The first time an event was issued. Used if the field "duration" is not NULL to implement this timing constraint

**Table 9:** The data dictionary of Conditions, a table used to facilitate testing the condition by dividing it into sub-conditions that are tested separately and then the final result is calculated from combining the sub-results with the appropriate logical operators

Column name	Data type	Constraints	Description
row_id	integer	Primary key, auto-increment	Sequence of object identifier
id	integer	Foreign key, Ref. table: allEvents, Ref. field: id	Serial number identifies the event
node_id	text	Allow null	Unique identifier for a sensing device in a specific land. Can be NULL if the table record stores the information of a logical operator from the tested condition
is_operator_record	integer	{0,1}	Can be 0 to indicate that the table record stores the information of a sub-condition from the tested condition, or 1 to indicate that the table record stores the information of a logical operator from the tested condition
subcondition	text		Relational sub-condition from the tested condition
result	boolean		The result of testing the sub-condition
